# Classification of gene signatures for their information value and functional redundancy

**DOI:** 10.1038/s41540-017-0038-8

**Published:** 2017-12-19

**Authors:** Laura Cantini, Laurence Calzone, Loredana Martignetti, Mattias Rydenfelt, Nils Blüthgen, Emmanuel Barillot, Andrei Zinovyev

**Affiliations:** 1 Institut Curie, PSL Research University, INSERM U900, Mines ParisTech, 26, rue d’Ulm, F-75248 Paris, France; 2 Institute of Pathology, Charite Universitätsmedizin Berlin, Chariteplatz 1, 10117 Berlin, Germany; 3 IRI Life Sciences and Institute for Theoretical Biology, Humboldt University, Philippstr. 13, Haus 18, 10115 Berlin, Germany

## Abstract

Gene signatures are more and more used to interpret results of omics data analyses but suffer from compositional (large overlap) and functional (correlated read-outs) redundancy. Moreover, many gene signatures rarely come out as significant in statistical tests. Based on pan-cancer data analysis, we construct a restricted set of 962 signatures defined as informative and demonstrate that they have a higher probability to appear enriched in comparative cancer studies. We show that the majority of informative signatures conserve their weights for the genes composing the signature (eigengenes) from one cancer type to another. We finally construct InfoSigMap, an interactive online map of these signatures and their cross-correlations. This map highlights the structure of compositional and functional redundancies between informative signatures, and it charts the territories of biological functions. InfoSigMap can be used to visualize the results of omics data analyses and suggests a rearrangement of existing gene sets.

## Data-driven signature classification

An informative collection of gene signatures for transcriptomic data analysis is constructed. The number of transcriptomic signatures grows fast and their collections are highly redundant that hampers omics data analyses interpretation. A computational biology team from Institut Curie led by Andrei Zinovyev selected a collection of 962 gene signatures shown to be informative for cancer studies and reflecting mechanisms of cancer progression. The signatures were filtered from a large compendium without requiring any manual curation by experts through a large-scale unbiased analysis of pancancer data. They have much higher chance to obtain significant enrichment scores in a comparative trancriptomic study. The authors integrated the 962 signatures into InfoSigMap, a new data visualization resource for the interpretation of the results of omics data analyses, which facilitates getting an insight into the mechanisms driving cancer.

## Introduction

**Fig. 1:**
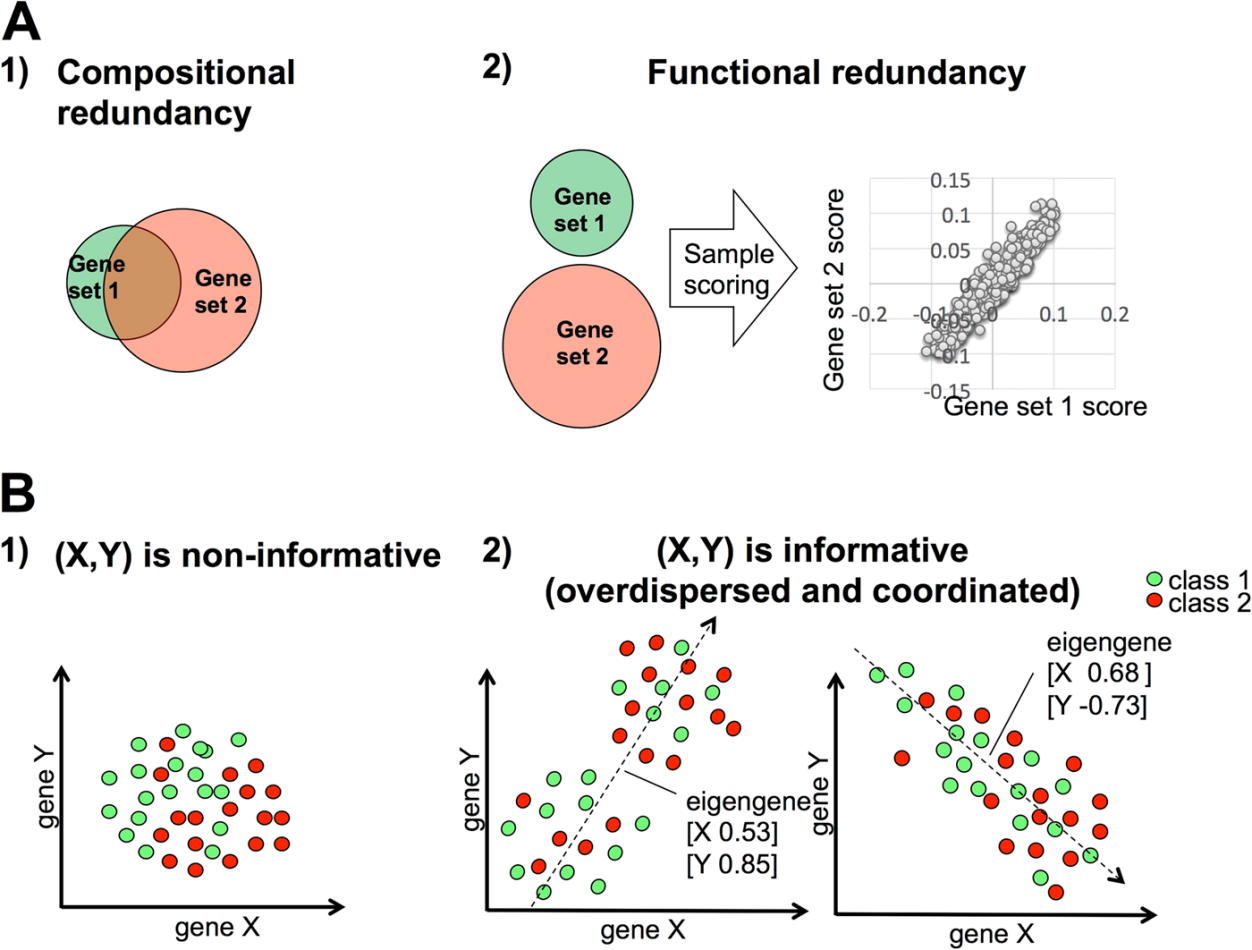
Schematic explanation of the basic notions used in this study. Panel **a** schematically summarizes the two possible forms of redundancy between two gene sets. (1) Compositional redundancy corresponds to gene set overlap. (2) Functional redundancy represents instead different transcriptional read-outs of the same biological process and it is possible even for the gene sets with no overlap. Measuring functional redundancy depends on the way a sample is scored based on the expression of its genes and the chosen corpus of data. Panel **b** explains the difference between non-informative (1) and informative (2) gene sets. A signature composed of two genes X and Y is here considered. The circles denote biological samples and the two colors correspond to two different labels: class 1 and class 2 (e.g., metastatic vs. primary tumors). Scatter plots are used to represent the expression values of gene X (*x*-axes) and gene Y (*y-*axes) in each sample. Three types of samples distributions are shown. In (1) (isotropic case), no naturally distinguished axis in the points' distribution, labeling of samples is needed to define their ranking. In (2) instead, it exists as a distinguishable axis in the data distribution that allows a robust ranking of the samples independently on their labeling. Both second and third scenario leads to overdispersion and coordination of the corresponding gene set and are selected in the analysis

The majority of the studies exploring gene expression data result in one or more gene signatures, i.e., list of genes sharing a common pattern of expression that can be employed to classify groups of samples in any independent dataset. Together with such data-derived signatures, a priori knowledge-based signatures can be produced from the available gene ontologies or pathway databases. In recent years, data–derived and a priori knowledge-based signatures have been widely employed to interpret the results of gene expression data analyses (e.g., differential expression, clustering). The number of available signatures is getting larger allowing users to benefit from a more exhaustive coverage of the existing biological processes. However, not all the signatures contained in these compendia are equally informative and the number of gene sets representing the same biological process is not equilibrated. These two phenomena affect the results of classical transcriptomic data analysis with heavy *p-*value corrections producing a high number of false negative results. Conceptually, the aforementioned gene set redundancy can be of two types: compositional or functional (see Fig. 1a). Compositionally redundant signatures are characterized by a large intersection in terms of the genes composing them. On the opposite, two signatures may represent two different transcriptional read-outs of the same biological process, we will refer to them as functionally redundant. These two types of redundancy do not always co-exist, in fact two signatures can be functionally redundant even if having no overlap. The existence of multiple functionally redundant signatures affects results of classical transcriptomic data analysis by highly scoring multiple gene sets belonging to analogous/related biological processes. These multiple comparisons of redundant signatures can potentially hide relevant hits. Of note, any estimation of the functional redundancy is conditioned by the context (e.g., certain cancer type) and therefore depends on the type of data used to evaluate the redundancy.

To our knowledge, few methods have been proposed to address the problem of gene signature redundancy.^1–6^ Currently, the best attempt to define a robust and non-redundant collection of signatures is represented by MSigDB Hallmarks (H).^7^ H was obtained by merging compositionally redundant signatures and then refining the genes of the resulting signatures based on their ability to discriminate the associated phenotype. This methodology involves a manual curation, which might create a certain bias vis a vis an expert’s opinion. More importantly, H, as all the other currently proposed procedures, takes into account only compositional redundancy without exploiting the problem of the functional one.

In this paper, a new approach to prioritize and classify gene signatures is proposed. Our method is based on the concept of “an informative signature”, which is a gene set capable of defining a natural ranking of samples in unsupervised way. Considering the simplest case of a gene signature composed of only two genes X and Y, their co-variance can define three possible scenarios of sample distribution (ranking), as reported in Fig. 1b. Whereas the ranking defined by informative signatures presents a distinguishable axis (corresponding to the direction of the first principal component), no naturally distinguished sample ranking can be observed in the non-informative ones. As a consequence, when the dysregulation of an informative signature is tested on a transcriptomic dataset whose samples are divided according to two conditions (e.g., tumor vs. normal), in most cases, a significant enrichment score will be observed whenever two requirements are met: (i) the direction of the largest variance sufficiently separates the samples belonging to the two conditions and (ii) this variance is significantly greater than randomly expected (the gene set is “overdispersed”). In all other cases, a very specific distribution of the sample labels is needed to obtain a significant enrichment score. Therefore, informative signatures, defining in many datasets robust and “objective” sample ranking along the principal variance direction, are more valuable for data analysis. Starting from a vast collection of signature compendia, composed of 12096 a priori knowledge and data-derived signatures, we defined a restricted set of 962 informative signatures, which is made available to the users for further applications. The collection was defined by exploiting various pan-cancer The Cancer Genome Atlas (TCGA) transcriptomic profiles (32 cancer types with totally 8991 samples). Among the databases under investigation, the Signaling Pathway Enrichment using Experimental Data sets (SPEED)^8^ signatures, including 11 gene sets, proved to be the most informative with 6 of them being part of our prioritized collection. The reliability of our signature collection was then validated by comparing its performances with those of the complete collection in some typical data analysis scenarios. In all the examples, the informative gene sets were found much more frequently significant than the others, confirming the rationale behind the selection procedure proposed here.

As a result of our analysis, in each dataset, a set of weights is also assigned to the genes composing the signatures. These weights correspond to the contributions of the genes to the direction of the first principal component and we refer to them as eigengene, according to the definition introduced by Langfelder et al.^9^ The eigengene associated with a signature can be used to compute the sample activity profile or metasample (see Methods section). We then define a signature “conserved” when its eigengenes are highly correlated across different cancer types. Here we found that informative signatures tend to be more conserved than the others, a further proof of the reliability of our collection. The collection of informative gene signatures was then classified by computing the average correlation between the sample activity profiles of all signatures across 32 cancers types. This metrics was used as a measure of functional redundancy in our analysis (see Methods section). We found that many signatures are functionally redundant and this does not seem to be always linked to the intersection between two gene sets. Therefore the H and the other previous works, only measuring the intersection in terms contained genes, are underestimating the scale of signature redundancy. In order to visually and interactively represent the structure of functional redundancies between informative gene signatures, we developed InfoSigMap (http://navicell.curie.fr/pages/maps_avcorrmodulenet.html), a user-friendly interactive Google Maps-based tool, where nodes correspond to our informative signatures and the edges represent the two types of redundancies (compositional and functional). InfoSigMap can be used for data visualization to provide a quick navigation into any set of scores associated with the informative signatures (e.g., enrichment scores), as shown here for some typical data analysis scenarios.

## Results

### Informative signatures represent a small fraction of the widely employed gene sets

**Fig. 2:**
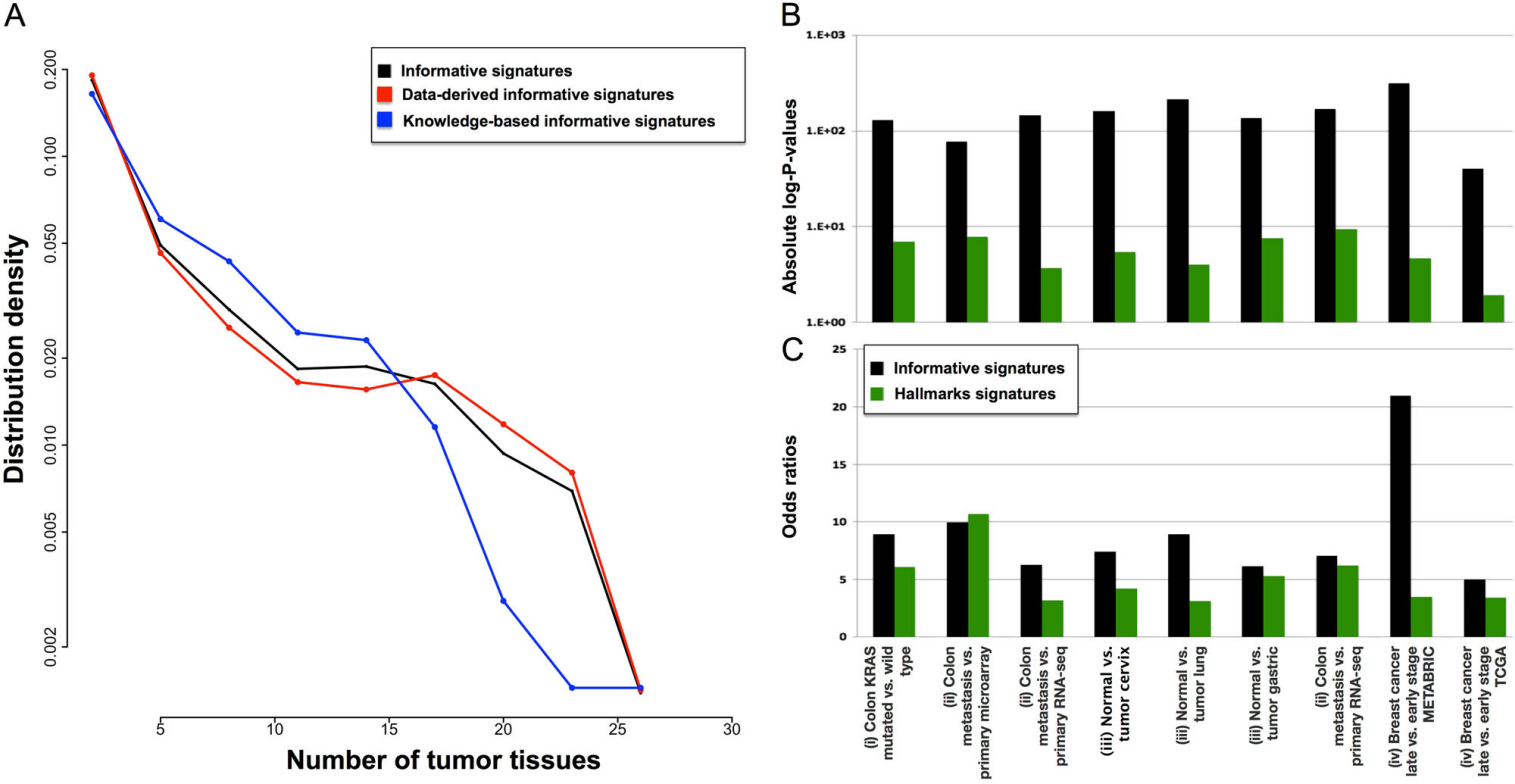
Properties of the informative signatures in different cancer types. In **a**, the distribution of the number of cancer types in which the informative signatures have ROMA L1 and L1/L2 *p*-values significant is reported (logarithmic scale). The behavior of all informative signatures, data-driven informative signatures, and knowledge-based informative signatures is represented in black, red, and blue, respectively. In **b** and **c**, the comparison between Informative (black) and Hallmarks (red) signatures is reported in all nine comparative tests cases. Enrichment of the corresponding subset (informative or Hallmarks) in the output of GSEA is evaluated in terms of Fisher absolute log-*p*-values and odds ratio, respectively

A large TCGA compendium of gene expression data derived from 32 solid cancer types was employed to restrict the input collection of 12,096 gene signatures to 962 (see Supplementary Table S2) informative ones (see Methods section). The selection involved the correction of *p*-values for multiple testing and the choice of stringent thresholds. To ensure a proper control of the False Discovery Rate (FDR), the number of false positive results of the performed analysis was estimated on 1000 random signatures (see Methods section). None of them resulted to be informative, according to the procedure here proposed. We thus estimate an FDR to less than 0.1%. Of the 962 identified informative signatures, the majority were data-derived (706 data-derived, 231 knowledge-based, 15 from MSigDB H collection, and 10 from MSigDB C1 collection), showing that for cancer-oriented applications, data-derived signatures tend to be better performing than knowledge-based ones. To assess which of the input compendia was more informative, the ratio between the number of informative gene sets and the total number of contained signatures was evaluated (Table 1). As shown in Table 1, the most informative compendium resulted to be SPEED (55% of informative signatures). The reliability of this database is thus corroborated by our results, suggesting that this collection particularly fits for cancer transcriptomic data analysis. Overall, good performances were also obtained by CIT (28%), the MSigDB C4 (31%), and the H (30%). The best performing knowledge-based database was Atlas of Cancer Signaling Network (ACSN), with 13% of informative signatures. Studying the distribution of the number of cancer types in which the signatures were found to be informative (Fig. 2a and Supplementary Table S2), we found that 30% of the informative signatures is cancer-specific (significant in only 2 cancer types), 14% are associated with >15 different cancer types and only 8 signatures are informative in >25 cancer types. Furthermore, data-driven signatures tend to be more frequently informative in multiple cancer types with respect to knowledge-based ones.

**Table 1:** Contribution of each signature collection to the informative set

Signatures collections	Data-derived						Knowledge-based				H	C1
	SPEED	C4	CIT	CGP	C7	C6	ACSN	CP	C5	C3		
Informative signatures	6	270	11	233	185	1	8	150	64	9	15	10
Total	11	858	40	1698	2436	99	63	1330	1454	615	50	326
Fraction	**55%**	**31%**	**28%**	14%	8%	1%	**13%**	11%	4%	1%	**30%**	3%

Number of informative signature, total dimension, and the fraction of the previous two fields are reported for each signature collection

Most informative signature collections are highlighted in bold

### Informative gene sets tend to be much more frequently significant in typical comparative cancer data analyses

Our hypothesis that an informative gene set has higher chances to be enriched in a typical transcriptomic data analysis, is tested here (see Methods section for the procedure) in four typical scenarios: (i) colorectal cancer KRAS mutated vs. wild type;^10^ (ii) colon cancer metastatic vs. primary (same samples profiled with two platforms: RNA–seq and microarrays);^11^ (iii) tumor vs. normal in four tissues (lung,^12^ gastric,^13^ colon,^14^ cervix^15^) and (iv) breast cancer late vs. early stage (from two independent data sources: TCGA and METABRIC^16^). As shown in Fig. 2b, in all four cases, the informative signatures in the output of the Gene Set Enrichment Analysis (GSEA)^17^ were strongly enriched (average *p*-value 10^−41^). Note that, while the selection of the informative signatures was performed using an unsupervised approach, the validations presented in this section are realized using a supervised one (GSEA). Nevertheless, the amount of informative signatures obtained in the output of the GSEA analysis is significantly higher than what could be expected at random. Finally, the results of points (ii) and (iv) prove that this analysis weakly depends on the platform used for transcriptomics profiling and it is well reproduced across independent datasets for the same biological case of study, respectively.

### Informative signatures perform better than the MSigDB Hallmarks in typical cancer data analysis

Given that the only other attempt to prioritize the most reliable non-redundant signatures is done by the MSigDB H, its performances were compared with those of our compendium in the four test cases previously mentioned. The comparison was done considering: Fisher’s exact test *p*-values, Fisher’s exact test odds ratios, and *p*-value of the Kolmogorov–Smirnov (KS) test for the Normalized Enrichment Score (NES) distributions (see Methods section). The *p*-values and odds ratios resulting from the Fisher’s exact test are summarized in Fig. 2b, c. As shown in the figure, the H signatures obtained significant Fisher *p*-value in all cases (average *p*-value 10^−5^), confirming its reliability. However, the *p*-values obtained by the H collection resulted to be always less significant than those of our informative signatures (10^−5^ vs. 10^−79^). Moreover, the Fisher’s exact test odds ratios for the informative signatures are higher than those of the H in eight out of the nine cases. Concerning the NES distribution, as shown in Supplementary Figure S1, the informative signatures tend to be always associated with absolute NES higher than those of the H. Indeed, the KS *p*-values are always <0.05, except for 2 out of the 9 cases. Therefore, not only the informative signatures are more frequently significant in a GSEA analysis but also in the GSEA output they tend to be among those with the highest NES score. This result indicates that our compendium is capturing the strongest sources of expression variation in all three transcriptomic datasets. As a further check, given that 15 out of the 50 H signatures are also contained in our informative collection, the fraction of H signatures present in both the output of the GSEA analysis and our informative compendium is evaluated: (i) colorectal cancer KRAS mutated vs. wild type 67%; (ii) colon cancer metastatic vs. primary 67 and 80% in RNA-seq and microarray, respectively; (iii) normal tissue vs. tumor in 4 tissues (lung 48%, gastric 47%, colon 44%, cervix 60%), and (iv) breast cancer late vs. early stage 33% TCGA and 87% METABRIC. These results show that, among the 50 signatures constituting the MSigDB Hallmarks, those that are found significant in the GSEA analysis are frequently also informative.

### The majority of the informative signature eigengenes are conserved across cancer types

**Fig. 3:**
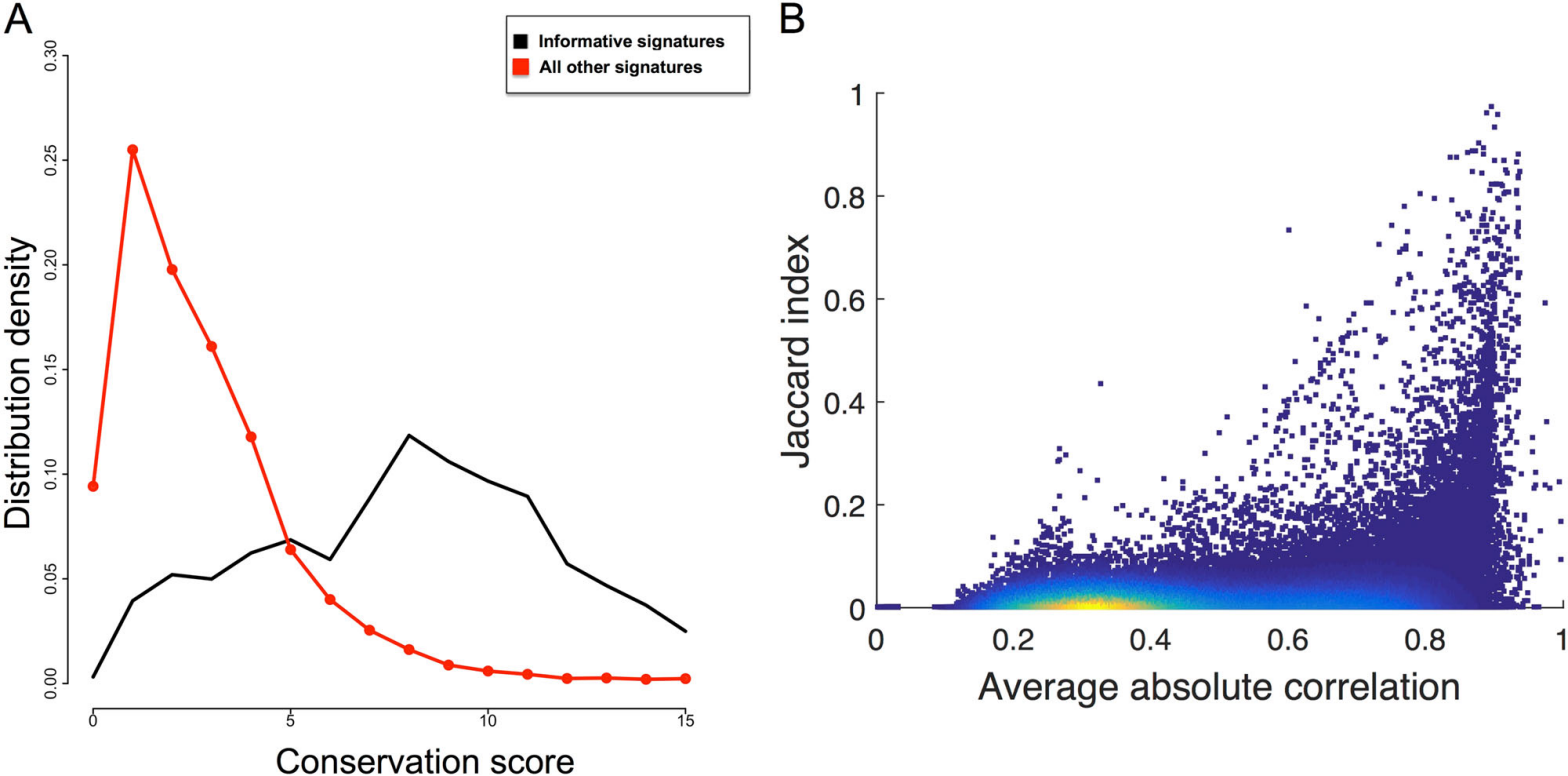
Pan-cancer correlations between the eigengenes of the informative signatures and the sample activity profiles. In **a**, the distribution of the conservation scores for the Informative (black) and the remaining signatures (red) is reported. In **b**, the dependence between informative gene sets overlap (Jaccard-index) and average correlation between the meta-samples defined by the informative gene sets is reported. Each point corresponds to an informative signature and their color is proportional to the point density: from red (high density) to blue (low density)

To further investigate the reliability of our informative collection, we verified whether the properties of the informative signatures were quantitatively reproduced across different cancer types. More precisely, we compared the eigengenes (set of gene weights) resulting from computing the first principal component restricted to the signature genes, across all 32 cancer types. We computed the conservation score as described in the Methods section. Then we compared the distribution of the conservation scores obtained for the informative signatures with those of the non-informative ones. As shown in Fig. 3a, informative signatures are associated with conservation scores higher than those of the other signatures. This distribution difference is significant with a KS *p*-value <10^−16^. Employing a rather restrictive threshold of 10^−6^ for the conservation score, 1459 over the 12,096 starting signatures (12%) resulted to be conserved across cancer types, whereas 703 over the 962 informative signatures (73%) were found to be conserved. This shows that the signatures selected with our approach have higher chances to maintain the same quantitative definition across different cancer types and thus they tend to be more robust than the starting ones.

### Functional redundancy of gene sets is poorly explained by their intersection size

Two gene sets with no intersection can represent the same biological process, being thus functionally redundant. For example, when data-derived signatures are constructed, only the genes whose expression is strongly associated with the phenotype of interest are kept. This procedure may lead to the reconstruction of two data-derived signatures associated with the same phenotype but having a poor/null intersection.^18^ To quantify the frequency of this phenomenon, the two redundancy measures: functional redundancy (computed as described in the Methods section) and computational redundancy (in terms of Jaccard-index (JI)) are compared (see Fig. 3b). As expected, the presence of a high JI value usually results in a high functional redundancy (i.e., high average correlation between meta-samples over all cancer types). However, high functional redundancy is distributed over a large range of JI values with a surprisingly higher density of points in the area corresponding to poorly overlapping gene sets. Therefore, in order to reduce the functional redundancy between gene sets, it is not only sufficient to simply take into account their overlap but it is important to consider also their correlation of activity. A first consequence of this result is that the H collection, based on the JI as a measure of redundancy, is not able to completely capture analogous signatures. The intrinsic limitation of such approach is that it requires the wide use of expression data and thus its output is at least partially data corpus-dependent.

### InfoSigMap a user-friendly interactive representation of the functional redundancy between informative signatures for insightful gene set score visualization

**Fig. 4:**
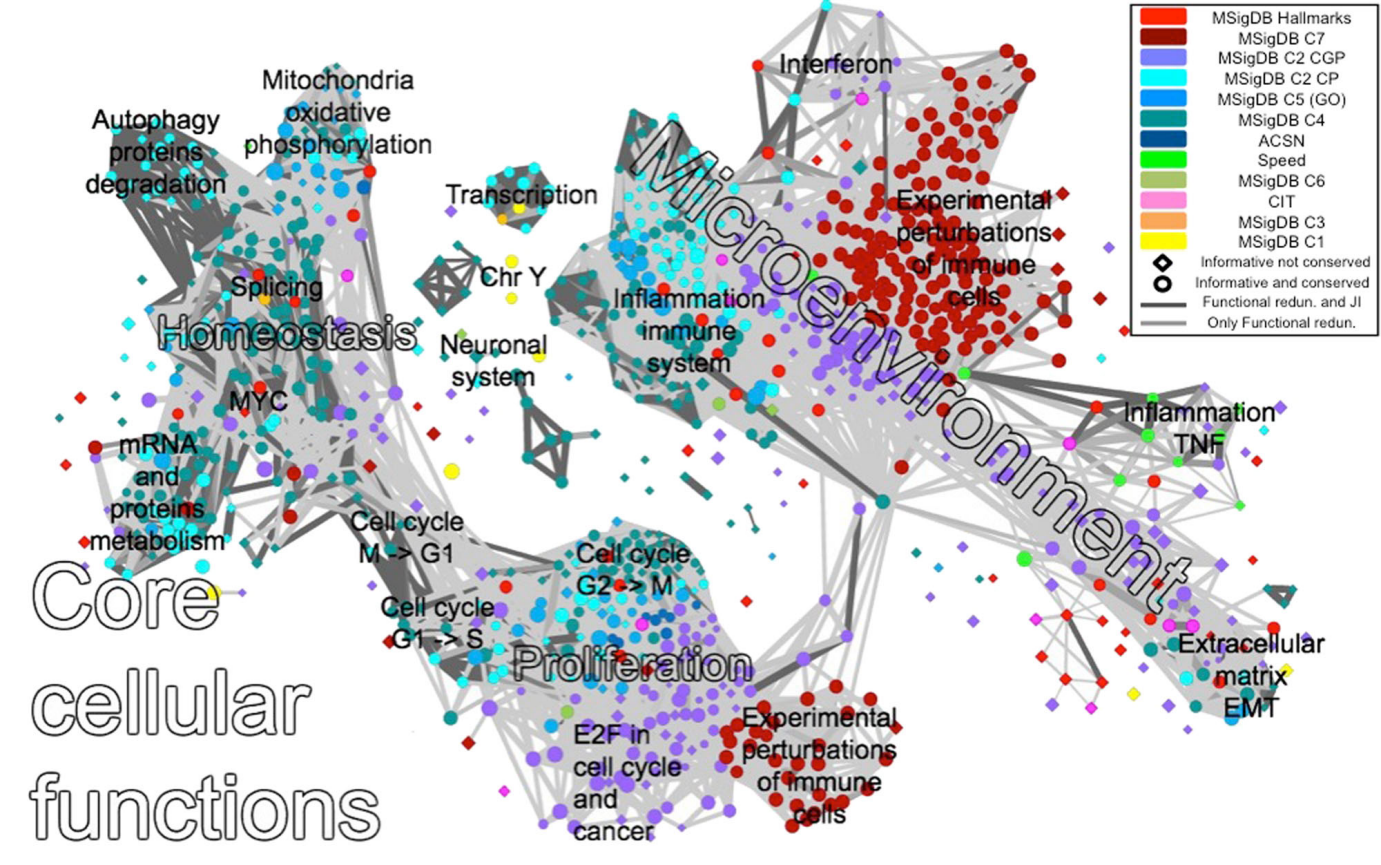
InfoSigMap: user-friendly interactive representation of the informative signatures and their redundancies. The network map of the 962 informative signatures plus SPEED and Hallmarks is here reported as available on the website (http://navicell.curie.fr/pages/maps_avcorrmodulenet.html). The signatures are organized as nodes of the network. Node colors correspond to the different signature categories, while the shape is a diamond for informative signatures and circular for those that are also conserved. The links correspond to redundancy between couples of gene sets (functional redundancy in light gray, dark gray if also the Jaccard–index intersection is significant). The names annotated on the top of the map denote areas of the network containing signatures associated with the same biological function. The interactive online version of this map can be browsed as an instance of Google Maps, with the possibility of zooming in and out, getting description of gene signatures, and visualizing data (various gene set scores, such as GSEA or ROMA scores) on top of the map

GSEA or alternative approaches can be used to score the informative signatures based on a transcriptomic dataset whose samples are divided according to multiple conditions. The output of such analyses usually consists of a table of gene sets with the corresponding enrichment values. This tabular organization of the output (which represents a one-dimensional ordering) is not easy to interpret and it frequently does not help the formulation of consistent biological hypothesis. To improve the interpretation, we developed InfoSigMap (http://navicell.curie.fr/pages/maps_avcorrmodulenet.html), following the procedure described in the Methods section. The obtained network (Fig. 4), whose nodes are the informative signatures and links denote their redundancy, contains eight connected components. The largest connected component is composed of two main clusters: one associated with core cellular functions (i.e., all those basic functions that are fundamental for the life of the cell) and the other to the tumor microenvironment. These two areas of the network can be then further subclustered. The core cellular functions can be split into homeostasis and proliferation (composed of cell cycle, mRNA translation, splicing, MYC targets, protein degradation, and oxidative phosphorylation). Of particular interest is the fact that, in this component, it is possible to clearly separate the signatures associated with the different cell cycle phases. The cluster of tumor microenvironment instead comprises: immune system, inflammation, tumor necrosis factor–α pathway, interferon, and extracellular matrix/epithelial–mesenchymal transition (EMT).

The map contains also other seven much smaller connected components. Among them, there are transcription, neuronal system, and some small components (not explicitly labeled on the map) that represent connections between genes systematically correlated in previous meta-analyses (GNF2^19^ and GCM^20^).

The two clusters constituting the largest connected component are connected through an area of signatures (dark red nodes on the map) associated with Experimental perturbations of Immune cells (EI). Indeed, the informative signatures derived from EI are split into two main areas (see Fig. 4): one belongs to the tumor microenvironment component and it is strongly linked to the immune system/inflammation signatures. The second is part of the proliferation component, strongly linked to the cell cycle area. We considered that such unexpected configuration could be caused by the presence of gene sets belonging to the EI category but obtained from the comparison of two conditions characterized by a strong difference in proliferation To test this hypothesis, the dataset used to define the MSigDB signature “LEE_NAIVE_T_LYMPHOCYTE”, obtained from the expression profiling of human CD4+ T cell during differentiation induction, was re-analyzed^21^ (see Supplementary Figure S2). The informative signatures differentially activated during the experiment (differentiated vs. undifferentiated states) were detected as described in the Methods section. The two areas of EI signatures show an opposite behavior coherent with that of the signatures around them. Indeed, the EI area near cell cycle shows a donwregulation in the differentiated cells, while the areas belonging to the immune cluster are upregulated. This confirms our starting hypothesis that, when EI signatures are used to analyze an expression dataset, their deregulation can be due to a variation in proliferation rather than in the immune system functioning. To avoid this uncertainty, a reorganization of the EI signatures (part of the MSigDB C7 collection) in several categories would be recommended for their future use in data analysis.

Another non-intuitive observation is that signatures coming from the same collection tend to co-localize in the map and data-derived signatures tend to be clearly separated from knowledge-based ones. The discrepancy between data-derived and knowledge-based signatures can be explained by the fact that the transcriptional readouts of a biological process might be very different from the genes involved in the process itself. Yet, another observation is that higher functional redundancy exists between signatures of the same collection rather than between signatures describing the same biological function. The only two exceptions to this trend, to some extent, are the MSigDB H collection and the SPEED signatures (although several non-informative SPEED signatures are clustered together). These compendia indeed resulted to be well spread around the map, confirming that they are able to efficiently capture the main biological signals encoded in the transcriptomic data. Nevertheless, some areas such as mRNA translation, transcription, splicing, and protein degradation were not covered by any of the H and SPEED signatures, indicating that other signatures are needed to have a complete portrait of the transcriptomic landscape.

InfoSigMap was developed to simplify the navigation and interpretation of the gene set score distributions. In the next section, some examples of typical analysis scenarios where InfoSigMap can be used to formulate consistent biological hypothesis are presented.

### InfoSigMap can be used to visualize the results of transcriptomic data analysis

**Fig. 5:**
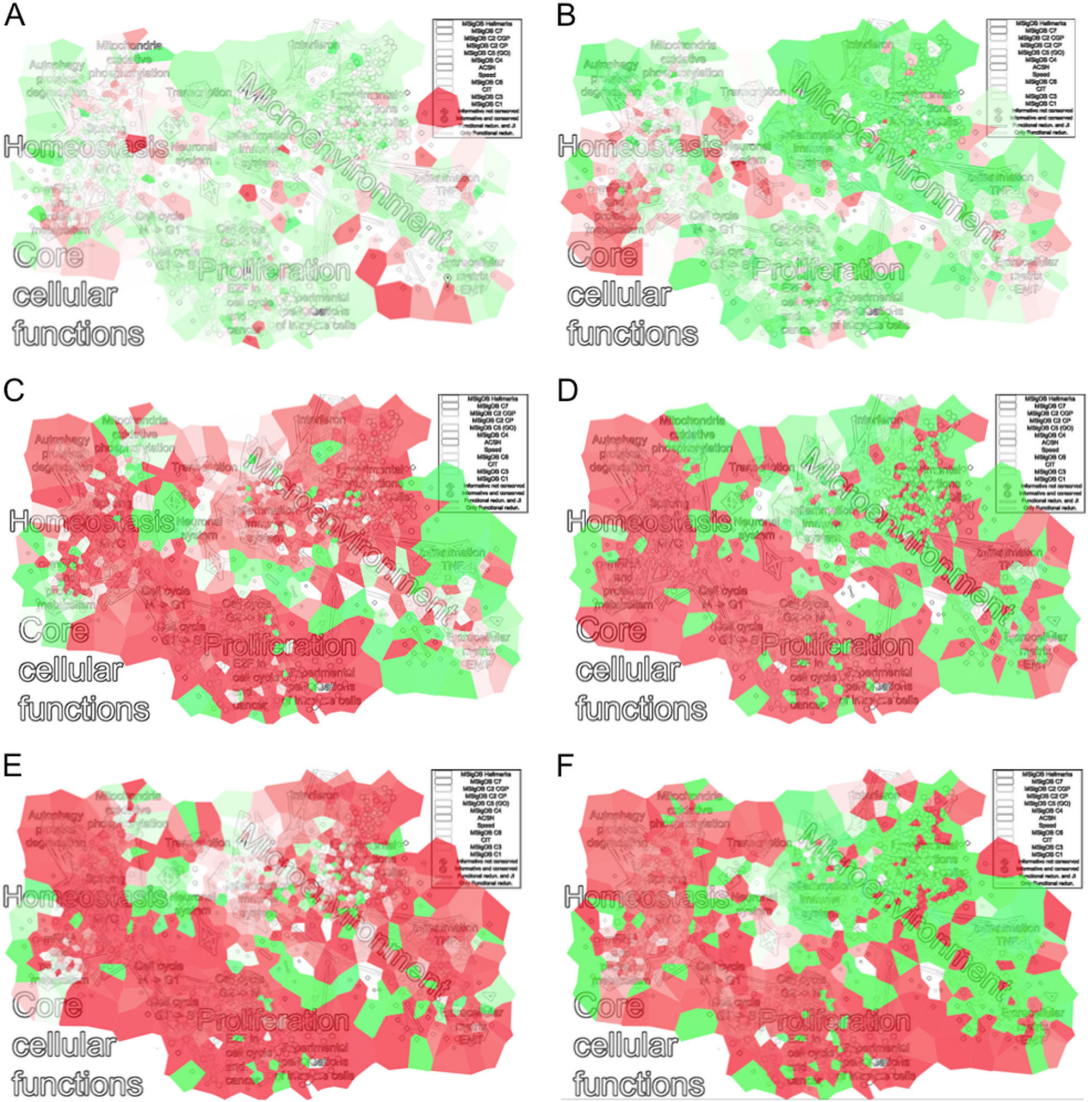
Results of InfoSigMap applied to some typical data analysis scenarios. Four examples showing how InfoSigMap provides an insightful interpretation of the lists of significantly enriched signatures, which are usually presented in a tabular form. The significant fold changes resulting from the differential ROMA analysis (see Methods section) are plotted on the top of the map according to a heatmap coloring highlighting upregulated (red) and downregulated (green) gene signatures. The layout of the map is the same in all six panels as in Fig. 4. The plots are organized as follows: **a** KRAS mutated vs. wild type colorectal cancer; **b** metastatic vs. primary colon cancer, and **c**–**f** tumor vs. normal tissue in cervix, colon, gastric, and lung, respectively

InfoSigMap is tested to investigate the alterations affecting the transcriptome of the three aforementioned typical cancer problems: (i) colorectal cancer KRAS mutated vs. wild type;^10^ (ii) colon cancer metastatic vs. primary (same samples profiled with two platforms: RNA-seq and microarrays);^11^ (iii) tumor vs. normal in four tissues (lung,^12^ gastric,^13^ colon,^14^ cervix^15^), and (iv) breast cancer late vs. early stage (from two data sources: TCGA and METABRIC^16^). The obtained results are shown in Fig. 5 and Supplementary Figure S3 (for the procedure, see Methods section). First, we show that the InfoSigMap profiles for the same type of analyses in the same cancer type stay the same, independently from data source (case iv) and platform (case ii) used (see Supplementary Figure S3). Then the biology of cases (i)-(iii) is discussed in detail:

(i) Colorectal cancer KRAS mutated vs. wild type:
  The impact of *KRAS* mutation on the transcriptome of colorectal cancer (CRC) is investigated with InfoSigMap (Fig. 5a). *KRAS* mutated CRC patients are known to be resistant to standard epidermal growth factor receptor (*EGFR*) inhibitory treatments.^22,23^ The output of our analysis can thus give some indications concerning possible new processes to be targeted in KRAS-mutated patients. The strongest effect reported in Fig. 5a (bright red area) is the upregulation of a subset of the metastatic signatures. This result fits with previous evidences that KRAS mutation is associated with metastasis in patients with CRC.^24,25^ Moreover an alteration of the metabolism is detectable from an upregulation of the mitochondria and oxidative phosphorylation areas. This result fits with previous experimental evidences. Indeed *KRAS* mutation has already been shown to induce mitochondrial oxidative stress, responsible for the so-called Warburg effect, a metabolic alteration fundamental for cancer cell proliferation.^26–28^ In CRC, *KRAS* mutation also causes an alteration of the transcriptional response and amino acid metabolism machineries, two processes involved in cancer cell proliferation and maintenance.^29,30^ This effect is captured in our analysis by the upregulation of the mRNA translation/protein metabolism areas of InfoSigMap.
(ii) Colon cancer metastatic vs. primary:
  The differential module activity between metastatic and primary colon cancer (CC) is investigated (Fig. 5b). As expected, an upregulation of the collagen/EMT area of the network clearly appears on the map. An upregulation of the miR-21 targets whose role in EMT is well known is also observed.^31,32^ Moreover, the areas: splicing, mRNA metabolism, and protein metabolism, resulted to be significantly upregulated. This is not surprising given that the aberration of the RNA-processing machinery (stability, metabolism, splicing, and polyadenylation) is known to be associated with cancer initiation and progression. In CC, beta-catenin (*CTNNB1*), involved in the Wnt pathway, is generally the cause of the RNA-processing alterations.^33–35^ This is confirmed in InfoSigMap; indeed *CTNNB1* is found active as shown by the upregulation of its targets (node FEVR_CTNNB1_TARGETS). The study of the cancer-specific RNA metabolism is a relatively unexplored area of research, with potentially significant implications for the prevention and treatment of CC. The above results confirm the experimentally observed *CTNNB1*-mediated alteration of the RNA-processing machinery. On the other side, a strong downregulation of the cells’ proliferative activity can be observed. This phenomenon has already been documented and found associated with poor prognosis in CRC.^36,37^ The observed slow proliferation in metastatic CC may be caused by a high proportion of cancer stem-like cells. Indeed, stem cells are in a quiescent state, which could explain the cell cycle downregulation identified in our analysis. The hypothesis of a high stem cell concentration is also confirmed by the significant downregulation of the immune area. Indeed, the stem-like phenotype of metastasis-initiating cells is generally associated with immune evasive quiescence, even if this point is not well documented in CC.^38^
(iii) Tumor vs. normal in four tissues: We then compare tumor vs. normal tissue in cervix, colon, gastric, and lung cancer (Fig. 5c–f). A global feature present in all four tissue types is the upregulation of the connected component associated with the core cellular functions. This is not a surprising result, since cancer cells generally inactivate tumor suppressors and hyperactivate oncogenes to promote sustained proliferation, alter autophagy and the various steps of the RNA transcription and translation processing machinery, develop metabolic imbalances, and enhance resistance to mitochondrial apoptosis.^39^ The microenvironment-associated connected component instead shows a dual behavior. It is significantly upregulated in cervical and gastric cancer and downregulated in the colon and lung. The results thus suggest a different role of the immune system in these four tumors. A possible explanation is that the tumors are associated with different levels of antigenicity, i.e., the extent to which tumor cells display HLA-restricted antigens that can be selectively or specifically recognized by T cells.^40^ Tumors with low antigenicity hide against cytotoxic attack leading to a passive escape from anti-cancer immune defense. This hypothesis is supported by the observation that the HLA signature in our network (GNF2_HLA_C) is concordantly downregulated in the lung and colon and upregulated in the cervix and gastric. Moreover, lung cancer association with low antigenicity has already been reported.^41^ The tumor antigenicity is one of the aspects that seem to determine whether a patient will respond to a given immunotherapy. A comprehensive pan-cancer classification of the immune component behavior could give indications regarding those individuals who are most likely to respond to immune-based therapies.

## Discussion

Data-driven and a priori knowledge-based gene signatures are largely used in cancer studies to score clinical samples according to distinct tumor subtypes, identify important cellular responses to stimuli, predict clinical outcomes, and quantify the activation of signaling pathways. Nowadays, signature collections are getting larger, providing the benefit of a more complete coverage of the existing biological processes. However, the growth of these compendia is posing two main challenges related to the reliability and the redundancy of the collected gene sets.

Here we developed a new methodology for assessing the value of a gene set, which is based on the notion of informative signature, i.e., a gene set able to systematically and robustly rank tumor samples in many independent datasets. A restricted collection of 962 informative gene sets is suggested for transcriptomic data analysis in cancer biology.

The methodology used for the selection of the informative signatures currently relies on Principal Component Analysis (PCA), a linear matrix factorization technique optimal for close-to-normal data distributions. In particular, the gene set overdispersion estimation is based on a linear model of gene regulation.^42^ For complex datasets, recapitulating many different and dynamically changing transcriptional programs, non-linear and strongly non-Gaussian features might play an important role. In this case, the suggested approach should be generalized by using, for example, principal curves, principal manifolds, or even more complicated branching data approximators.^43–45^ This might be particularly important in the case of exploiting the data coming from the application of single-cell sequencing technology.^46^ However, the concepts of informative gene signature and functional redundancy introduced in this study should be useful and instrumental independently on the concrete methodology used for quantifying them. In the current method, we exploit only the major direction of the variance, reflected in the first principal component; in the future, we will envisage considering two-dimensional modes of pathway activation, characterized by activity of two or even more hidden factors.

The robustness of the information content enclosed in our compendium is tested here on several scenarios of typical cancer problems. The results show that an informative gene set has much higher chances to be selected (enriched) in a typical scenario of transcriptomic data analyses, even in the ones using supervised methods, and that the eigengenes of the majority of the informative signatures tend to be conserved across cancer types. The redundancy of the informative collection is then investigated, showing that functional redundancy is a frequent phenomenon not captured by the previously proposed approaches. We developed InfoSigMap, a user-friendly interface designed for insightful data visualization. The advantage of using our map with respect to the classical tabular outputs of GSEA or analogous tools is proved by the wide use of pre-existing tools with a similar aim (e.g., Enrichment Map, GOIorize, and ClueGO). At the same time, InfoSigMap has multiple novelties with respect to those tools. It is a map of informative signatures and not only of Gene Ontology (GO) categories. Moreover, a link between two signatures in InfoSigMap reflects the existence of a functional redundancy between the two gene sets, never considered before.

The NaviCell-based implementation of InfoSigMap allows easy integration of our tool in any pipeline for omics data analysis (from Java, R, or Python programming languages) using simple REST API. InfoSigMap is already used for the interpretation of Independent Component Analysis results in the BIODICA software (https://github.com/LabBandSB/BIODICA) and of cBioPortal data using NaviCom web-service.^47^

InfoSigMap was applied on some typical scenarios of cancer data analysis. The obtained results showed that a global view of the concordant behavior of functionally redundant signatures leads to an insightful interpretation with respect to what can be deduced from simple lists of significant signatures. In all four analyzed cases, the obtained results were found to fit with the previous experimental knowledge, confirming the reliability of our approach. However, also some indications concerning new candidate mechanisms to be experimentally investigated were extracted, showing how InfoSigMap can help in the formulation of new biological hypothesis.

## Methods

### Definition of “an informative signature”

PCA is applied to a gene expression data table where columns correspond to the genes from a selected gene set and where rows correspond to samples. If the variance explained by the first principal component computed for such a table is significantly larger than for a random set of genes of the same size then the considered gene set is called overdispersed. Intuitively, an overdispersed gene set has a stronger contribution to the data variance than expected by chance. Similarly, if the ratio between the variances explained by the first and second principal components computed for the aforementioned table is larger than for a random set of genes, then the given gene set is called coordinated. Intuitively, the existence of a statistically significant gap between the first and the second eigenvalue of the covariance matrix corresponds to an overall increase in the pairwise correlations between the genes of the signature and what can be observed at random. The advantage of having a coordinated gene set is that it defines an axis of principal variance in the multi-dimensional distribution of samples and thus robustly ranks samples independently on the group to which the samples belong (see Fig. 1). In the context of cancer biology, we define informative a gene set that is simultaneously overdispersed and coordinated in more than two cancer types.

### Transcriptomics data used in our analysis

To systematically search for informative signatures, a large pan-cancer TCGA compendium of gene expression data derived from 32 solid cancer types (ACC, BLCA, BRCA, CESC, CHOL, COAD, DLBC, ESCA, GBM, HNSC, KICH, KIRC, KIRP, LGG, LIHC, LUAD, LUSC, MESO, OV, PAAD, PCPG, PRAD, READ, SARC, SKCM, STAD, TGCT, THCA, THYM, UCEC, UCS, UVM) was used. The data were downloaded from TCGA and normalized. An overview of the samples available for the different tumor types is reported in Supplementary Table S1.

### Collections of signatures used in the analysis

A vast collection composed of both data-derived and a priori knowledge-based signatures was considered as input for our analysis. The signature collections: Molecular Signature Database (MsigDB v5.2),^17^ ACSN,^48^ the top ontributing genes of the components identified by Biton et al. (here denoted as CIT),^49^ and the SPEED,^8^ have been downloaded, obtaining a complete collection of 12,096 signatures. We consider as data-derived signatures: CIT, SPEED, and some MSigDB categories (clusters of genes co-expressed in microarray compendia (C4), signatures of oncogenic pathway activation (C6), the large collection of immunological conditions (C7), and chemical and genetic perturbations (CGP) part of the MSigDB collection canonical pathways and experimental signatures curated from publications (C2)). We consider as knowledge-based signatures: ACSN and the MSigDB categories: genes sharing cis-regulatory motifs upstream or downstream of their coding sequence (C3), genes grouped according to GO categories (C5), and canonical pathways, including the well-known BIOCARTA, KEGG, and REACTOME databases. The MSigDB collections genes grouped by their location in the human genome (C1) and the H are not associated with any of the two previous classifications.

### Procedure for the prioritization of those signatures that are informative in cancer biology

To detect which of the starting 12,096 signatures were informative, we employed the Representation and quantification Of Module Activity (ROMA) tool, designed for the robust detection of overdispersed and coordinated gene sets.^42^ The activity of each signature was thus evaluated in all the 32, previously described, expression datasets separately. We considered two p–values output of ROMA: the first one associated with the variance explained by the first principal component (ROMA L1 score) and the second one associated with the ratio between the variances explained by the first and second principal components (ROMA L1/L2 score). The two *p*-values have been corrected for multiple testing through Benjamini–Hochberg method. We will thus denote in the following with P_FDRs the corrected *p*-values. Only those signatures having the two P_FDRs <0.05 in at least two tumor datasets were prioritized. To evaluate the number of false positives produced by our analysis, we designed a null model constituted by 1000 random signatures. These signatures have been built by sampling the size distribution of the starting 12,096 signatures and by extracting random genes for each one of the size percentiles. The genes for the null model have been randomly picked from the list of all the genes contained in the 12,096 signatures maintaining repetitions. Therefore, if a gene was present in 100 signatures in the original collection, it is present in multiple signatures also in the null model. We are thus preserving signatures dependences at least to some extent. The procedure described above to detect informative signatures was applied to this collection of 1000 random signatures. None of these random signatures was found informative. Finally, we tested whether our analysis was biased by the use of TCGA data. Six hundred and ninety-two out of the 962 signatures remain informative for non-TCGA cancer datasets (colon 98 samples, cervix 33 samples, lung 58 samples, gastric 38 samples, and METABRIC breast cancer 1454 samples) (Supplementary Table S2) Note that the non-TCGA datasets, except that of METABRIC, are smaller (in terms of samples) than the TCGA ones originally used for detecting informative signatures.

### Test of the informative signatures in typical cancer analysis scenarios

Our hypothesis that an informative gene set is more likely to be enriched in a typical transcriptomic data analysis, is tested by using GSEA,^17^ a well-known and widely adopted supervised approach.^17^ GSEA was applied to three typical cancer-related examples using the entire collection of 12,096 signatures. The set of significant signatures was determined selecting those with a GSEA FDR *q*-value <0.05. The statistical significance of the number of informative signatures present in the output of the GSEA analysis was evaluated through a Fisher’s exact test.

### Comparison of Informative signatures vs. Hallmarks

The procedure described in the previous section was repeated also for the MSigDB H. The performances of our informative collection were then compared with those of the H using both Fisher’s exact test *p*–values and odds ratio. The distributions of the absolute GSEA NES for the two collections were studied, and the significance of the difference between the two distributions was evaluated through KS test.

### Evaluation of the signatures eigengenes conservation across cancers

Accorginly to the definition introduced in ref. ^9^, eigengene is one of the right singular vectors of the gene expression matrix. Considering the singular value decomposition of a gene expression matrix (gene vs. sample) *X* = *USV*, eigengene is a row vector from matrix *V*, corresponding to the largest value on the diagonal of *S*. The metasample or sample activity profile is the *U* vector, corresponding to the largest value on the diagonal of *S*. The eigengenes associated with the informative signatures have been computed on all the 32 analyzed datasets (as a result of the PCA). For each informative signature, the pair-wise correlation between the eigengenes obtained in the 32 cancer types were computed and a conservation score was obtained as the absolute logarithm of the geometric mean of the Pearson correlation *p*-values.

### Comparison between functional redundancy and intersection size of gene sets

For each couple of gene sets, we have compared their normalized intersection size (JI) vs. their functional redundancy. The functional redundancy for a couple of gene sets was measured according to a two steps process. First, Pearson correlation coefficients between the metasamples associated to the two signatures were computed in each of the 32 cancer datasets. We thus obtained 32 correlation scores for each couple of signatures. Then to summarize them, their average was computed.

### InfoSigMap construction procedure

The construction of InfoSigMap involved three main steps: (i) creation of the signature redundancy graph; (ii) definition of its layout, and (iii) representation of the graph as an interactive online map. In line with what has been already done in Enrichment Map, GOIorize, and ClueGO,^2,4,6^ the first step is performed by organizing the 990 signatures (corresponding to the 962 informative collection plus all H and SPEED signatures even if they were not shown to be informative) into a weighted network, where each signature is a node and links represent redundancy between couples of gene sets. Differently from the previously mentioned Cytoscape plug-ins, the links of our network are weighted averaging over two measures of signatures redundancy: compositional (JI) and functional redundancy (computed as described above). A link is present between two nodes only if the corresponding signatures have a functional redundancy >0.7. This threshold is justified by appearance of distinguishable but still connected functional components in the graph. For the second step of graph structure representation, a different shape is used to denote the gene sets that are only informative (diamond) and those that are also conserved (circle), while the node size denotes the number of genes in the signature. Links are also classified into two classes, dark gray is used for those edges that connect signatures being both functionally redundant and having a significant JI, while light gray denotes links only associated with functional redundancy. Finally, the thickness of the links is proportional to their weights, the standard Cytoscape organic layout is used to spatially organize the largest connected component of the network and smaller components or unconnected nodes were positioned by using the structure of weaker correlations. The areas of the network containing signatures associated with same biological functions are then identified and manually annotated on the top of the map to help the navigation of the users. In addition, also a purely data-driven layout was computed by applying tSNE dimension reduction method to the matrix of average pairwise correlations between the meta-samples defined by our signatures in all cancer types. This view of the InfoSigMap is available at http://navicell.curie.fr/pages/maps_avcorrmodulenet.html (View/tSNE selection in the right-hand panel). Finally, the representation of the network as an interactive online map is achieved by using NaviCell,^50^ powered by Google Maps API.

### Using InfoSigMap to have a global view of the signatures behavior

Gene sets can be tested for differential activity across different experimental conditions using a tool of choice (e.g., GSEA, ROMA). Here the activity of the informative signatures is evaluated by applying ROMA, then the differential module activity is evaluated by Student’s *t*-test, and fold change applied to the ROMA activity scores. Finally, the fold changes associated with a significant Student’s *t*-test *p*-value (<0.05) are mapped to the nodes of InfoSigMap as a color gradient, from red (upregulation) to white (no significant change) to green (downregulation), using the map staining approach described in ref. ^50^ The map is thus colored in the territories around each node creating a continuous colored pattern that helps a qualitative appreciation of the concordant/discordant behavior of large map regions.

### Data availability

The pan-cancer transcriptomic datasets used in this study are freely available on the TCGA data portal https://portal.gdc.cancer.gov. The InfoSigMap tool together with the.gmt file of the prioritized signatures are available at http://navicell.curie.fr/pages/maps_avcorrmodulenet.html. The tools and resources used in this study are publicly available. Custom codes used in the study are available upon request.

## Electronic supplementary material

Supplementary Informations

## Electronic supplementary material

**Supplementary information** accompanies the paper on the *npj Systems Biology and Applications* website (10.1038/s41540-017-0038-8).
