# Supplementary material for: Classification of gene signatures for their information value and functional redundancy
Source: NPJ Syst Biol Appl. 2017 Dec 19;4:2. doi: 10.1038/s41540-017-0038-8 (PMC5736638; doi:10.1038/s41540-017-0038-8)
Supplement: Supplementary file 1 — Supplementary Informations [file 41540_2017_38_MOESM1_ESM.pdf]

Supplementary Information

Classification of gene signatures for their information value and functional redundancy

Laura Cantini[1], Laurence Calzone[1], Loredana Martignetti[1], Mattias Rydenfelt[2,3], Nils Blüthgen[2,3], Emmanuel Barillot[1], Andrei Zinovyev[1]

1. Institut Curie, PSL Research University, INSERM U900, Mines ParisTech, 26, rue d'Ulm, F-75248 Paris, France.
2. Institute of Pathology, Charite Universitätsmedizin Berlin, Chariteplatz 1, 10117 Berlin, Germany.
3. IRI Life Sciences and Institute for Theoretical Biology, Humboldt University, Philippstr. 13, Haus 18, 10115 Berlin, Germany

Correspondence : Laura Cantini (laura.cantini@curie.fr) and Andrei Zinovyev (andrei.zinovyev@curie.fr)

Supplementary contents

Table S1. Number of samples available for each transcriptomic dataset used for the analysis.

Table S2. List of informative signatures and number of tumors in which they were found significant.

Figure S1. Informative vs. Hallmarks GSEA NES score distributions comparison.

Figure S2. ROMA activity score on human CD4+ T cell during differentiation plotted on InfoSigMap

Figure S3. InfoSigMap profiles are independent from the data source and platform used.

Table S1. Number of samples available for each transcriptomic dataset used for the analysis

tumor code	tumor name extended	number of samples
ACC	Adrenocortical carcinoma	78
BLCA	Bladder Urothelial Carcinoma	408
BRCA	Breast invasive carcinoma	1085
CESC	Cervical squamous cell carcinoma and endocervical adenocarcinoma	303
CHOL	Cholangiocarcinoma	36
COAD	Colon adenocarcinoma	276
DLBC	Lymphoid Neoplasm Diffuse Large B-cell Lymphoma	48
ESCA	Esophageal carcinoma	182
GBM	Glioblastoma multiforme	155
HNSC	Head and Neck squamous cell carcinoma	515
KICH	Kidney Chromophobe	65
KIRC	Kidney renal clear cell carcinoma	516
KIRP	Kidney renal papillary cell carcinoma	286
LGG	Brain Lower Grade Glioma	513
LIHC	Liver hepatocellular carcinoma	368
LUAD	Lung adenocarcinoma	510
LUSC	Lung squamous cell carcinoma	488
MESO	Mesothelioma	87
OV	Ovarian serous cystadenocarcinoma	302
PAAD	Pancreatic adenocarcinoma	177
PCPG	Pheochromocytoma and Paraganglioma	181
PRAD	Prostate adenocarcinoma	494
READ	Rectum adenocarcinoma	90
SARC	Sarcoma	255
SKCM	Skin Cutaneous Melanoma	103
STAD	Stomach adenocarcinoma	401
TGCT	Testicular Germ Cell Tumors	138
THCA	Thyroid carcinoma	501
THYM	Thymoma	120
UCEC	Uterine Corpus Endometrial Carcinoma	173
UCS	Uterine Carcinosarcoma	57
UVM	Uveal Melanoma	80

Table S2. List of informative signatures and number of cancer types in which they were found significant

Signature	Compendium of origin	Tumors in which is informative	Informative in non-TCGA datasets
REACTOME_RESPIRATORY_ELECTRON_TRANSPORT_ATP_SYNTHESIS_BY_CHEMIOSMOTIC_COUPLING_AND_HEAT_PRODUCTION_BY_UNCOUPLING_PROTEINS	MSigDB C2 CP	28	1
CIT8_UP	CIT	27	1
GNF2_EIF3S6	MSigDB C4	27	1
MORF_TPT1	MSigDB C4	27	1
CIT8_DN	CIT	26	1
GNF2_CCNB2	MSigDB C4	26	1
MORF_ACTG1	MSigDB C4	26	1
REACTOME_RESPIRATORY_ELECTRON_TRANSPORT	MSigDB C2 CP	26	1
CIT7_DN	CIT	25	1
GNF2_BUB1B	MSigDB C4	25	1
GNF2_CDC20	MSigDB C4	25	1
GNF2_HMMR	MSigDB C4	25	1
GNF2_PCNA	MSigDB C4	25	1
GNF2_ST13	MSigDB C4	25	1
KOBAYASHI_EGFR_SIGLING_6HR	MSigDB C2 CGP	25	1
MORF_NPM1	MSigDB C4	25	1
chryq11	MSigDB C1	24	1
GCM_NPM1	MSigDB C4	24	1
GNF2_CCNA2	MSigDB C4	24	1
GNF2_FBL	MSigDB C4	24	1
GNF2_GLTSCR2	MSigDB C4	24	1
GNF2_RRM2	MSigDB C4	24	1
HALLMARK_ALLOGRAFT_REJECTION	MSigDB H	24	1
MORF_NME2	MSigDB C4	24	1
FARMER_BREAST_CANCER_CLUSTER_2	MSigDB C2 CGP	23	1
GNF2_CASP1	MSigDB C4	23	1
GNF2_CD7	MSigDB C4	23	1
GNF2_CENPE	MSigDB C4	23	1
GNF2_MCM4	MSigDB C4	23	1
GNF2_TTK	MSigDB C4	23	1
MORF_RAN	MSigDB C4	23	1
GCM_TPT1	MSigDB C4	22	1
GNF2_CDC2	MSigDB C4	22	1
GNF2_CKS2	MSigDB C4	22	1
GNF2_FEN1	MSigDB C4	22	1
MORF_AP2M1	MSigDB C4	22	1
MORF_GPX4	MSigDB C4	22	1
REACTOME_METABOLISM_OF_MRNA	MSigDB C2 CP	22	1
REACTOME_METABOLISM_OF_RNA	MSigDB C2 CP	22	1
MODULE_22	MSigDB C4	21	0
GNF2_BUB1	MSigDB C4	21	1
GNF2_DAP3	MSigDB C4	21	1
GNF2_HCK	MSigDB C4	21	1
GNF2_MKI67	MSigDB C4	21	1
GNF2_S100A4	MSigDB C4	21	1
MORF_CSNK2B	MSigDB C4	21	1
MORF_EIF3S6	MSigDB C4	21	1
MORF_ERH	MSigDB C4	21	1
MORF_MAP2K2	MSigDB C4	21	1
MORF_PPP1CA	MSigDB C4	21	1
MORF_UBE2I	MSigDB C4	21	1
GNF2_UBE2I	MSigDB C4	20	0
CHANG_CYCLING_GENES	MSigDB C2 CGP	20	1
GNF2_CENPF	MSigDB C4	20	1
GNF2_RRM1	MSigDB C4	20	1
GNF2_SMC4L1	MSigDB C4	20	1
GNF2_TNFRSF1B	MSigDB C4	20	1
GNF2_TPT1	MSigDB C4	20	1
HALLMARK_OXIDATIVE_PHOSPHORYLATION	MSigDB H	20	1
KEGG_ALLOGRAFT_REJECTION	MSigDB C2 CP	20	1
KEGG_TYPE_I_DIABETES_MELLITUS	MSigDB C2 CP	20	1
MODULE_84	MSigDB C4	20	1
MORF_SOD1	MSigDB C4	20	1
REACTOME_TRANSLATION	MSigDB C2 CP	20	1
WALLACE_PROSTATE_CANCER_RACE	MSigDB C2 CGP	20	1
ZHOU_CELL_CYCLE_GENES_IN_IR_RESPONSE_6HR	MSigDB C2 CGP	20	1
MODULE_83	MSigDB C4	19	1
GNF2_CKS1B	MSigDB C4	19	1
GNF2_ESPL1	MSigDB C4	19	1
GNF2_SMC2L1	MSigDB C4	19	1
KEGG_AUTOIMMUNE_THYROID_DISEASE	MSigDB C2 CP	19	1
KEGG_OXIDATIVE_PHOSPHORYLATION	MSigDB C2 CP	19	1
KONG_E2F1_TARGETS	MSigDB C2 CGP	19	1
MCLACHLAN_DENTAL_CARIES	MSigDB C2 CGP	19	1
MODULE_54	MSigDB C4	19	1
TURASHVILI_BREAST_CARCINOMA_DUCTAL_VS_LOBULAR	MSigDB C2 CGP	19	1
ZHANG_TLX_TARGETS_60HR	MSigDB C2 CGP	19	1
chryp11	MSigDB C1	18	0
HOFMANN_MYELODYSPLASTIC_SYNDROM_RISK	MSigDB C2 CGP	18	1
CIT12_UP	CIT	18	1
CROONQUIST_IL6_DEPRIVATION	MSigDB C2 CGP	18	1
GCM_ACTG1	MSigDB C4	18	1
GCM_CSNK2B	MSigDB C4	18	1
GNF2_CARD15	MSigDB C4	18	1
GNF2_ITGB2	MSigDB C4	18	1
GNF2_PECAM1	MSigDB C4	18	1
GNF2_PTPN4	MSigDB C4	18	1
GNF2_RFC3	MSigDB C4	18	1
GNF2_RFC4	MSigDB C4	18	1
GUTIERREZ_WALDENSTROEMS_MACROGLOBULINEMIA_2	MSigDB C2 CGP	18	1
MODULE_152	MSigDB C4	18	1
MODULE_62	MSigDB C4	18	1
MORF_EIF3S2	MSigDB C4	18	1
MORF_FBL	MSigDB C4	18	1
MORF_G22P1	MSigDB C4	18	1
REACTOME_3_UTR_MEDIATED_TRANSLATIONAL_REGULATION	MSigDB C2 CP	18	1
REACTOME_INFLUENZA_VIRAL_RNA_TRANSCRIPTION_AND_REPLICATION	MSigDB C2 CP	18	1
REACTOME_SRP_DEPENDENT_COTRANSLATIONAL_PROTEIN_TARGETING_TO_MEMBRANE	MSigDB C2 CP	18	1
MODULE_91	MSigDB C4	17	1
BIOCARTA_CTLA4_PATHWAY	MSigDB C2 CP	17	1
GNF2_CD1D	MSigDB C4	17	1
GNF2_H2AFX	MSigDB C4	17	1
GNF2_IL2RB	MSigDB C4	17	1
GSE10325_LUPUS_CD4_TCELL_VS_LUPUS_MYELOID	MSigDB C7	17	1
HALLMARK_E2F_TARGETS	MSigDB H	17	1
HALLMARK_G2M_CHECKPOINT	MSigDB H	17	1
HALLMARK_INTERFERON_GAMMA_RESPONSE	MSigDB H	17	1
KEGG_GRAFT_VERSUS_HOST_DISEASE	MSigDB C2 CP	17	1
LEE_DIFFERENTIATING_T_LYMPHOCYTE	MSigDB C2 CGP	17	1
MITOCHONDRIAL_PART	MSigDB C5	17	1
MORF_ANP32B	MSigDB C4	17	1
MORF_BUB1	MSigDB C4	17	1
MORF_PPP2R4	MSigDB C4	17	1
MORF_PSMC1	MSigDB C4	17	1
REACTOME_IMMUNOREGULATORY_INTERACTIONS_BETWEEN_A_LYMPHOID_AND_A_NON_LYMPHOID_CELL	MSigDB C2 CP	17	1
REACTOME_INFLUENZA_LIFE_CYCLE	MSigDB C2 CP	17	1
REACTOME_TCA_CYCLE_AND_RESPIRATORY_ELECTRON_TRANSPORT	MSigDB C2 CP	17	1
REACTOME_TCR_SIGNALING	MSigDB C2 CP	17	1
SCHUETZ_BREAST_CANCER_DUCTAL_INVASIVE	MSigDB C2 CGP	17	1
GSE22886_NAIVE_CD8_TCELL_VS_MONOCYTE	MSigDB C7	16	0
REACTOME_COSTIMULATION_BY_THE_CD28_FAMILY	MSigDB C2 CP	16	0

CIT3_UP	CIT	16	1
GCM_APEX1	MSigDB C4	16	1
GNF2_CD97	MSigDB C4	16	1
HALLMARK_EPITHELIAL_MESENCHYMAL_TRANSITION	MSigDB H	16	1
HALLMARK_INTERFERON_ALPHA_RESPONSE	MSigDB H	16	1
KEGG_ANTIGEN_PROCESSING_AND_PRESENTATION	MSigDB C2 CP	16	1
MODULE_47	MSigDB C4	16	1
MORF_ATOX1	MSigDB C4	16	1
MORF_DAP3	MSigDB C4	16	1
MORF_EIF4A2	MSigDB C4	16	1
REACTOME_MRNA_SPLICING_MINOR_PATHWAY	MSigDB C2 CP	16	1
REACTOME_NONSENSE_MEDIATED_DECAY_ENHANCED_BY_THE_EXON_JUNCTION_COMPLEX	MSigDB C2 CP	16	1
REACTOME_PEPTIDE_CHAIN_ELONGATION	MSigDB C2 CP	16	1
TURASHVILI_BREAST_LOBULAR_CARCINOMA_VS_LOBULAR_NORMAL	MSigDB C2 CGP	16	1
MODULE_114	MSigDB C4	15	0
MODULE_42	MSigDB C4	15	1
BOSCO_TH1_CYTOTOXIC_MODULE	MSigDB C2 CGP	15	1
E2F4_TARGETS	ACSN	15	1
GNF2_ICAM3	MSigDB C4	15	1
GNF2_MCL1	MSigDB C4	15	1
GNF2_MYD88	MSigDB C4	15	1
GSE10325_CD4_TCELL_VS_MYELOID	MSigDB C7	15	1
GSE10325_LUPUS_CD4_TCELL_VS_LUPUS_BCELL	MSigDB C7	15	1
GSE22886_NAIVE_CD4_TCELL_VS_MONOCYTE	MSigDB C7	15	1
KEGG_INTESTINAL_IMMUNE_NETWORK_FOR_IGA_PRODUCTION	MSigDB C2 CP	15	1
MODULE_151	MSigDB C4	15	1
MORF_HDAC1	MSigDB C4	15	1
REACTOME_G2_M_CHECKPOINTS	MSigDB C2 CP	15	1
REACTOME_VIF_MEDIATED_DEGRADATION_OF_APOBEC3G	MSigDB C2 CP	15	1
RPS14_DN.V1	MSigDB C2 CGP	15	1
SHIPP_DLBCL_CURED_VS_FATAL	MSigDB C2 CGP	15	1
CHUNG_BLISTER_CYTOTOXICITY	MSigDB C2 CGP	14	0
IMMUNE_SYSTEM_PROCESS	MSigDB C5	14	1
GNF2_CD33	MSigDB C4	14	1
GNF2_MATK	MSigDB C4	14	1
GNF2_PTPRC	MSigDB C4	14	1
GNF2_ZAP70	MSigDB C4	14	1
GRAHAM_NORMAL_QUIESCENT_VS_NORMAL_DIVIDING	MSigDB C2 CGP	14	1
GSE10325_CD4_TCELL_VS_BCELL	MSigDB C7	14	1
MODULE_45	MSigDB C4	14	1
MORF_BUB1B	MSigDB C4	14	1
PID_PLK1_PATHWAY	MSigDB C2 CP	14	1
PID_TCR_PATHWAY	MSigDB C2 CP	14	1
POMEROY_MEDULLOBLASTOMA_DESMOPLASIC_VS_CLASSIC	MSigDB C2 CGP	14	1
PUJA_BREAST_CANCER_LIT_INT_NETWORK	MSigDB C2 CGP	14	1
SMID_BREAST_CANCER_NORMAL_LIKE	MSigDB C2 CGP	14	1
SPINDLE	MSigDB C5	14	1
VILIMAS_NOTCH1_TARGETS	MSigDB C2 CGP	14	1
MODULE_46	MSigDB C4	13	1
MODULE_75	MSigDB C4	13	1
MODULE_77	MSigDB C4	13	1
REACTOME_SCF_BETA_TRCP_MEDIATED_DEGRADATION_OF_EMI1	MSigDB C2 CP	13	1
GNF2_ITGAL	MSigDB C4	13	1
GNF2_MCM5	MSigDB C4	13	1
GNF2_SELL	MSigDB C4	13	1
GSE10325_BCELL_VS_MYELOID	MSigDB C7	13	1
GSE15750_DAY6_VS_DAY10_EFF_CD8_TCELL	MSigDB C7	13	1
GSE22886_NAIVE_TCELL_VS_MONOCYTE	MSigDB C7	13	1
HALLMARK_MYC_TARGETS_V1	MSigDB H	13	1
JISON_SICKLE_CELL_DISEASE	MSigDB C2 CGP	13	1
KEGG_RIBOSOME	MSigDB C2 CP	13	1
MODULE_292	MSigDB C4	13	1
MODULE_57	MSigDB C4	13	1
MORF_JUND	MSigDB C4	13	1
REACTOME_ACTIVATION_OF_ATR_IN_RESPONSE_TO_REPLICATION_STRESS	MSigDB C2 CP	13	1
REACTOME_CELL_CYCLE_MITOTIC	MSigDB C2 CP	13	1
REACTOME_RNA_POL_I_PROMOTER_OPENING	MSigDB C2 CP	13	1
WONG_MITOCHONDRIA_GENE_MODULE	MSigDB C2 CGP	13	1
JAATINEN_HEMATOPOIETIC_STEM_CELL	MSigDB C2 CGP	12	1
KOBAYASHI_EGFR_SIGLING_24HR	MSigDB C2 CGP	12	0
S_CC_PHASE	ACSN	12	0
BIOCARTA_NO2IL12_PATHWAY	MSigDB C2 CP	12	1
E2F3_TARGETS	ACSN	12	1
GNF2_FGR	MSigDB C4	12	1
GNF2_HLA-C	MSigDB C4	12	1
GSE15750_DAY6_VS_DAY10_TRAF6KO_EFF_CD8_TCELL	MSigDB C7	12	1
M_PHASE	MSigDB C5	12	1
MITOCHONDRIAL_MEMBRANE_PART	MSigDB C5	12	1
MORF_DEK	MSigDB C4	12	1
MORF_SKP1A	MSigDB C4	12	1
PID_IL12_2PATHWAY	MSigDB C2 CP	12	1
REACTOME_ACTIVATION_OF_THE_PRE_REPLICATIVE_COMPLEX	MSigDB C2 CP	12	1
REACTOME_MITOTIC_M_M_G1_PHASES	MSigDB C2 CP	12	1
UROSEVIC_RESPONSE_TO_IMIQUIMOD	MSigDB C2 CGP	12	1
CHOI_ATL_CHRONIC_VS_ACUTE	MSigDB C2 CGP	11	0
DEFENSE_RESPONSE	MSigDB C5	11	0
GAZDA_DIAMOND_BLACKFAN_ANEMIA_PROGENITOR	MSigDB C3	11	0
GSE11057_PBMC_VS_MEM_CD4_TCELL	MSigDB C7	11	0
HOFFMAN_CLOCK_TARGETS	MSigDB C2 CGP	11	1
REACTOME_FORMATION_OF_ATP_BY_CHEMIOSMOTIC_COUPLING	MSigDB C2 CP	11	0
T_CELL_ACTIVATION	MSigDB C5	11	1
BILANGES_SERUM_AND_RAPAMYCIN_SENSITIVE_GENES	MSigDB C2 CGP	11	1
FIK_BREAST_CANCER_SDPP_SIGTURE	MSigDB C2 CGP	11	1
GAURNIER_PSMD4_TARGETS	MSigDB C2 CGP	11	1
GNF2_CD53	MSigDB C4	11	1
GSE40685_TREG_VS_FOXP3_KO_TREG_PRECURSOR	MSigDB C7	11	1
IMMUNE_RESPONSE	MSigDB C5	11	1
KEGG_PARKINSONS_DISEASE	MSigDB C2 CP	11	1
MODULE_171	MSigDB C4	11	1
MORF_BUB3	MSigDB C4	11	1
MORF_CTBP1	MSigDB C4	11	1
MORF_PPP6C	MSigDB C4	11	1
MORF_RAD23A	MSigDB C4	11	1
PID_CD8_TCR_PATHWAY	MSigDB C2 CP	11	1
REACTOME_CDK_MEDIATED_PHOSPHORYLATION_AND_REMOVAL_OF_CDC6	MSigDB C2 CP	11	1
REACTOME_DNA_REPLICATION	MSigDB C2 CP	11	1
REACTOME_GENERATION_OF_SECOND_MESSENGER_MOLECULES	MSigDB C2 CP	11	1
REACTOME_PD1_SIGNALING	MSigDB C2 CP	11	1
SA_RESPONSE_TO_IFNG	MSigDB C2 CGP	11	1
TURASHVILI_BREAST_NORMAL_DUCTAL_VS_LOBULAR	MSigDB C2 CGP	11	1
ZHAN_MULTIPLE_MYELOMA	MSigDB C2 CGP	11	1
HALLMARK_INFLAMMATORY_RESPONSE	MSigDB H	10	0
ORGANELLAR_RIBOSOME	MSigDB C5	10	1
PID_AURORA_B_PATHWAY	MSigDB C2 CP	10	1
PIEPOLI_LGI1_TARGETS	MSigDB C2 CGP	10	1
RUTELLA_RESPONSE_TO_HGF	MSigDB C2 CGP	10	0
WEE	ACSN	10	1
GCM_PPP1CC	MSigDB C4	10	1
GCM_PSME1	MSigDB C4	10	1
GNF2_TNFSF10	MSigDB C4	10	1
GSE19888_ADENOSINE_A3R_INH_PRETREAT_AND_ACT_BY_A3R_VS_TCELL_MEMBRANES_ACT_MAST_CELL	MSigDB C7	10	1
HOLLEMAN_ASPARAGISE_RESISTANCE_ALL	MSigDB C2 CGP	10	1
ICHIBA_GRAFT_VERSUS_HOST_DISEASE_D7	MSigDB C2 CGP	10	1
JIANG_HYPOXIA_VIA_VHL	MSigDB C2 CGP	10	1
KAUFFMANN_MELANOMA_RELAPSE	MSigDB C2 CGP	10	1
KEGG_CELL_CYCLE	MSigDB C2 CP	10	1
KEGG_SPLICEOSOME	MSigDB C2 CP	10	1
MODULE_158	MSigDB C4	10	1

MODULE_208	MSigDB C4	10	1
MODULE_44	MSigDB C4	10	1
MORF_EI24	MSigDB C4	10	1
MORF_RAD21	MSigDB C4	10	1
MORF_SART1	MSigDB C4	10	1
REACTOME_AUTODEGRADATION_OF_THE_E3_UBIQUITIN_LIGASE_COP1	MSigDB C2 CP	10	1
REACTOME_MITOTIC_PROMETAPHASE	MSigDB C2 CP	10	1
REACTOME_PHOSPHORYLATION_OF_CD3_AND_TCR_ZETA_CHAINS	MSigDB C2 CP	10	1
RUTELLA_RESPONSE_TO_HGF_VS_CSF2RB_AND_IL4	MSigDB C2 CGP	10	1
GSE24634_TREG_VS_TCONV_POST_DAY10_IL4_CONVERSION	MSigDB C7	9	0
HSIAO_HOUSEKEEPING_GENES	MSigDB C2 CGP	9	1
KEGG_CYTOKINE_CYTOKINE_RECEPTOR_INTERACTION	MSigDB C2 CP	9	1
MARSON_FOXP3_TARGETS	MSigDB C2 CGP	9	0
MITOCHONDRIAL_ENVELOPE	MSigDB C5	9	0
MORF_CCNI	MSigDB C4	9	0
QI_PLASMACYTOMA	MSigDB C2 CGP	9	0
REACTOME_DESTABILIZATION_OF_MRNA_BY_AUF1_HNRNP_D0	MSigDB C2 CP	9	0
RIBONUCLEOPROTEIN_COMPLEX	MSigDB C5	9	1
RIBOSOME	MSigDB C5	9	1
RUTELLA_RESPONSE_TO_CSF2RB_AND_IL4	MSigDB C2 CGP	9	0
BENPORATH_PROLIFERATION	MSigDB C2 CGP	9	1
BOWIE_RESPONSE_TO_EXTRACELLULAR_MATRIX	MSigDB C2 CGP	9	1
CROONQUIST_NRAS_SIGLING	MSigDB C2 CGP	9	1
GNF2_CDH11	MSigDB C4	9	1
GNF2_INPP5D	MSigDB C4	9	1
GNF2_NPM1	MSigDB C4	9	1
GNF2_RAB7L1	MSigDB C4	9	1
GSE10325_LUPUS_BCELL_VS_LUPUS_MYELOID	MSigDB C7	9	1
GSE14415_TCONV_VS_FOXP3_KO_INDUCED_TREG	MSigDB C7	9	1
GSE21063_WT_VS_NFATC1_KO_8H_ANTI_IGM_STIM_BCELL	MSigDB C7	9	1
GSE29618_PDC_VS_MDC_DAY7_FLU_VACCINE	MSigDB C7	9	1
GSE37533_PPARG1_FOXP3_VS_FOXP3_TRANSDUCED_CD4_TCELL	MSigDB C7	9	1
HORTON_SREBF_TARGETS	MSigDB C2 CGP	9	1
M_PHASE_OF_MITOTIC_CELL_CYCLE	MSigDB C5	9	1
MITOCHONDRIAL_MEMBRANE	MSigDB C5	9	1
MITOCHONDRION	MSigDB C5	9	1
MORF_AP3D1	MSigDB C4	9	1
MORF_RAD23B	MSigDB C4	9	1
REACTOME_CDT1_ASSOCIATION_WITH_THE_CDC6_ORC_ORIGIN_COMPLEX	MSigDB C2 CP	9	1
REACTOME_DNA_STRAND_ELONGATION	MSigDB C2 CP	9	1
REACTOME_P53_INDEPENDENT_G1_S_DNA_DAMAGE_CHECKPOINT	MSigDB C2 CP	9	1
GRAHAM_CML_DIVIDING_VS_NORMAL_DIVIDING	MSigDB C2 CGP	8	0
GSE22886_NAIVE_BCELL_VS_NEUTROPHIL	MSigDB C7	8	0
KEGG_HUNTINGTONS_DISEASE	MSigDB C2 CP	8	1
LI_WILMS_TUMOR_APLASTIC	MSigDB C2 CGP	8	0
LYMPHOCYTE_ACTIVATION	MSigDB C5	8	0
MITOCHONDRIAL_INNER_MEMBRANE	MSigDB C5	8	0
MITOCHONDRIAL_RESPIRATORY_CHAIN	MSigDB C5	8	0
MODULE_103	MSigDB C4	8	0
MODULE_388	MSigDB C4	8	1
PID_ATR_PATHWAY	MSigDB C2 CP	8	1
REACTOME_INTERFERON_ALPHA_BETA_SIGNALING	MSigDB C2 CP	8	0
REACTOME_REGULATION_OF_ORNITHINE_DECARBOXYLASE_ODC	MSigDB C2 CP	8	1
REACTOME_SIGNALING_BY_WNT	MSigDB C2 CP	8	1
YAO_TEMPORAL_RESPONSE_TO_PROGESTERONE_CLUSTER_12	MSigDB C2 CGP	8	0
BROWNE_INTERFERON_RESPONSIVE_GENES	MSigDB C2 CGP	8	1
E2F6_TARGETS	ACSN	8	1
GNF2_APEX1	MSigDB C4	8	1
GNF2_PA2G4	MSigDB C4	8	1
GNF2_PTPN6	MSigDB C4	8	1
GNF2_STAT6	MSigDB C4	8	1
GSE29618_BCELL_VS_MONOCYTE	MSigDB C7	8	1
GSE3982_MEMORY_CD4_TCELL_VS_BCELL	MSigDB C7	8	1
KEGG_NATURAL_KILLER_CELL_MEDIATED_CYTOTOXICITY	MSigDB C2 CP	8	1
KEGG_PROTEASOME	MSigDB C2 CP	8	1
MODULE_125	MSigDB C4	8	1
MODULE_27	MSigDB C4	8	1
MODULE_28	MSigDB C4	8	1
MODULE_32	MSigDB C4	8	1
MODULE_403	MSigDB C4	8	1
MORF_ESPL1	MSigDB C4	8	1
MORF_PRDX3	MSigDB C4	8	1
MORF_PRKAR1A	MSigDB C4	8	1
MORF_PSMC2	MSigDB C4	8	1
MORF_RRM1	MSigDB C4	8	1
MORF_XRCC5	MSigDB C4	8	1
PICCALUGA_ANGIOIMMUNOBLASTIC_LYMPHOMA	MSigDB C2 CGP	8	1
PID_IL12_STAT4_PATHWAY	MSigDB C2 CP	8	1
REACTOME_MITOCHONDRIAL_PROTEIN_IMPORT	MSigDB C2 CP	8	1
REACTOME_MRNA_SPLICING	MSigDB C2 CP	8	1
RODWELL_AGING_KIDNEY	MSigDB C2 CGP	8	1
RORIE_TARGETS_OF_EWSR1_FLI1_FUSION	MSigDB C2 CGP	8	1
WEBER_METHYLATED_LCP_IN_SPERM	MSigDB C2 CGP	8	1
BIOCARTA_IL12_PATHWAY	MSigDB C2 CP	7	0
CIT14_UP	CIT	7	0
FULCHER_INFLAMMATORY_RESPONSE_LECTIN_VS_LPS	MSigDB C2 CGP	7	0
GRADE_METASTASIS	MSigDB C2 CGP	7	0
GSE22140_HEALTHY_VS_ARTHRITIC_GERMFREE_MOUSE_CD4_TCELL	MSigDB C7	7	0
KEGG_CELL_ADHESION_MOLECULES_CAMS	MSigDB C2 CP	7	1
MATTIOLI_MULTIPLE_MYELOMA_WITH_14Q32_TRANSLOCATIONS	MSigDB C2 CGP	7	0
MITOCHONDRIAL_RIBOSOME	MSigDB C5	7	0
MOLEAR_TARGETS_OF_CCND1_AND_CDK4	MSigDB C2 CGP	7	1
MORF_RAB5A	MSigDB C4	7	0
ONO_AML1_TARGETS	MSigDB C2 CGP	7	1
POSITIVE_REGULATION_OF_IMMUNE_SYSTEM_PROCESS	MSigDB C5	7	0
REACTOME_AUTODEGRADATION_OF_CDH1_BY_CDH1_APC_C	MSigDB C2 CP	7	0
REACTOME_INTERFERON_GAMMA_SIGNALING	MSigDB C2 CP	7	0
BIOCARTA_PROTEASOME_PATHWAY	MSigDB C2 CP	7	1
CELL_CYCLE_PROCESS	MSigDB C5	7	1
FUJII_YBX1_TARGETS	MSigDB C2 CGP	7	1
GABRIELY_MIR21_TARGETS	MSigDB C2 CGP	7	1
GNF2_FOS	MSigDB C4	7	1
GNF2_G22P1	MSigDB C4	7	1
GNF2_MSH2	MSigDB C4	7	1
GNF2_RAN	MSigDB C4	7	1
GNF2_VAV1	MSigDB C4	7	1
GSE13485_CTRL_VS_DAY7_YF17D_VACCINE_PBMC	MSigDB C7	7	1
GSE13547_CTRL_VS_ANTI_IGM_STIM_BCELL_12H	MSigDB C7	7	1
GSE14415_INDUCED_VS_NATURAL_TREG	MSigDB C7	7	1
GSE22140_GERMFREE_VS_SPF_MOUSE_CD4_TCELL	MSigDB C7	7	1
GSE29618_MONOCYTE_VS_PDC	MSigDB C7	7	1
GSE3039_NKT_CELL_VS_ALPHAALPHA_CD8_TCELL	MSigDB C7	7	1
GSE39556_CD8A_DC_VS_NK_CELL_MOUSE_3H_POST_POLYIC_INJ_UP	MSigDB C7	7	1
GSE42724_NAIVE_BCELL_VS_PLASMABLAST	MSigDB C7	7	1
HALLMARK_TNFA_SIGNALING_VIA_NFKB	MSigDB H	7	1
KEGG_LEISHMANIA_INFECTION	MSigDB C2 CP	7	1
MITOSIS	MSigDB C5	7	1
MITOTIC_CELL_CYCLE	MSigDB C5	7	1
MODULE_128	MSigDB C4	7	1
MODULE_170	MSigDB C4	7	1
MODULE_252	MSigDB C4	7	1
MODULE_315	MSigDB C4	7	1
MODULE_345	MSigDB C4	7	1
MORF_FEN1	MSigDB C4	7	1
MORF_RFC4	MSigDB C4	7	1
MORF_UNG	MSigDB C4	7	1
MORI_IMMATURE_B_LYMPHOCYTE	MSigDB C2 CGP	7	1
REACTOME_CELL_CYCLE	MSigDB C2 CP	7	1

REACTOME_G1_S_TRANSITION	MSigDB C2 CP	7	1
REACTOME_PACKAGING_OF_TELOMERE_ENDS	MSigDB C2 CP	7	1
STRUCTURAL_CONSTITUENT_OF_RIBOSOME	MSigDB C5	7	1
AMUNDSON_GAMMA_RADIATION_RESISTANCE	MSigDB C2 CGP	6	0
BOYLAN_MULTIPLE_MYELOMA_PCA3	MSigDB C2 CGP	6	0
BROWN_MYELOID_CELL_DEVELOPMENT	MSigDB C2 CGP	6	0
CELL_CYCLE_CHECKPOINT_GO_0000075	MSigDB C5	6	0
chr5q31	MSigDB C1	6	0
DIAZ_CHRONIC_MEYLOGENOUS_LEUKEMIA	MSigDB C2 CGP	6	0
GSE14000_UNSTIM_VS_4H_LPS_DC	MSigDB C7	6	0
GSE21360_NAIVE_VS_QUATERNARY_MEMORY_CD8_TCELL	MSigDB C7	6	0
GSE21360_PRIMARY_VS_TERTIARY_MEMORY_CD8_TCELL	MSigDB C7	6	0
GSE22140_GERMFREE_VS_SPF_ARTHRITIC_MOUSE_CD4_TCELL	MSigDB C7	6	0
GSE29618_BCELL_VS_MDC_DAY7_FLU_VACCINE	MSigDB C7	6	0
GSE29618_MONOCYTE_VS_MDC	MSigDB C7	6	0
GSE30962_PRIMARY_VS_SECONDARY_ACUTE_LCMV_INF_CD8_TCELL	MSigDB C7	6	0
GSE37532_WT_VS_PPARG_KO_VISCERAL_ADIPOSE_TISSUE_TREG	MSigDB C7	6	0
GSE7509_UNSTIM_VS_IFNA_STIM_IMMATURE_DC	MSigDB C7	6	0
HOFFMANN_PRE_BI_TO_LARGE_PRE_BII_LYMPHOCYTE	MSigDB C2 CGP	6	1
HONMA_DOCETAXEL_RESISTANCE	MSigDB C2 CGP	6	1
MODULE_307	MSigDB C4	6	1
REACTOME_INTERFERON_SIGNALING	MSigDB C2 CP	6	0
REACTOME_OLFACTORY_SIGNALING_PATHWAY	MSigDB C2 CP	6	0
STARK_PREFRONTAL_CORTEX_22Q11_DELETION	MSigDB C2 CGP	6	1
TSUTSUMI_FBXW8_TARGETS	MSigDB C2 CGP	6	1
ASTASSIOU_CANCER_MESENCHYMAL_TRANSITION_SIGTURE	MSigDB C2 CGP	6	1
BIOCARTA_CTL_PATHWAY	MSigDB C2 CP	6	1
BIOCARTA_TCYTOTOXIC_PATHWAY	MSigDB C2 CP	6	1
CELL_CYCLE_PHASE	MSigDB C5	6	1
CHROMOSOME	MSigDB C5	6	1
DISTECHE_ESCAPED_FROM_X_ICTIVATION	MSigDB C2 CGP	6	1
FERREIRA_EWINGS_SARCOMA_UNSTABLE_VS_STABLE	MSigDB C2 CGP	6	1
GNF2_CD14	MSigDB C4	6	1
GOLDRATH_ANTIGEN_RESPONSE	MSigDB C2 CGP	6	1
GSE7218_UNSTIM_VS_ANTIGEN_STIM_THROUGH_IGG_BCELL	MSigDB C7	6	1
IL-1	Speed	6	1
KEGG_HEMATOPOIETIC_CELL_LINEAGE	MSigDB C2 CP	6	1
LINDGREN_BLADDER_CANCER_CLUSTER_2B	MSigDB C2 CGP	6	1
MALO_HYPOXIA	MSigDB C2 CGP	6	1
MODULE_1	MSigDB C4	6	1
MODULE_198	MSigDB C4	6	1
MODULE_436	MSigDB C4	6	1
MODULE_79	MSigDB C4	6	1
MOOTHA_HUMAN_MITODB_6_2002	MSigDB C2 CGP	6	1
MORF_AATF	MSigDB C4	6	1
MORF_ACP1	MSigDB C4	6	1
MORF_GSPT1	MSigDB C4	6	1
MORF_HAT1	MSigDB C4	6	1
MORF_HDAC2	MSigDB C4	6	1
MORF_UBE2N	MSigDB C4	6	1
NABA_CORE_MATRISOME	MSigDB C2 CP	6	1
REACTOME_G1_S_SPECIFIC_TRANSCRIPTION	MSigDB C2 CP	6	1
REACTOME_MITOTIC_G1_G1_S_PHASES	MSigDB C2 CP	6	1
RNA_BINDING	MSigDB C5	6	1
SPINDLE_MICROTUBULE	MSigDB C5	6	1
DAVICIONI_MOLECULAR_ARMS_VS_ERMS	MSigDB C2 CGP	5	0
GSE23568_CTRL_TRANSDUCED_VS_WT_CD8_TCELL	MSigDB C7	5	0
GSE29618_MONOCYTE_VS_MDC_DAY7_FLU_VACCINE	MSigDB C7	5	0
GSE37533_PPARG1_FOXP3_VS_PPARG2_FOXP3_TRANSDUCED_CD4_TCELL	MSigDB C7	5	0
JAK-STAT	Speed	5	1
KEGG_CHEMOKINE_SIGNALING_PATHWAY	MSigDB C2 CP	5	1
KIM_RESPONSE_TO_TSA_AND_DECITABINE	MSigDB C2 CGP	5	0
KRCTCNNNNMANAGC_UNKNOWN	MSigDB C3	5	0
LEOUR_DENDRITIC_CELL_MATURATION	MSigDB C2 CGP	5	0
MITOCHONDRIAL_LUMEN	MSigDB C5	5	0
MITOCHONDRIAL_RESPIRATORY_CHAIN_COMPLEX_I	MSigDB C5	5	0
MODULE_124	MSigDB C4	5	0
MODULE_273	MSigDB C4	5	1
REACTOME_IMMUNE_SYSTEM	MSigDB C2 CP	5	0
REACTOME_MEIOSIS	MSigDB C2 CP	5	0
REACTOME_METABOLISM_OF_PROTEINS	MSigDB C2 CP	5	0
REACTOME_RNA_POL_I_RNA_POL_III_AND_MITOCHONDRIAL_TRANSCRIPTION	MSigDB C2 CP	5	1
RIBOSOMAL_SUBUNIT	MSigDB C5	5	1
TAKEDA_TARGETS_OF_NUP98_HOXA9_FUSION_8D	MSigDB C2 CGP	5	1
TLR	Speed	5	0
TNFa	Speed	5	0
BIOCARTA_TCAPOPTOSIS_PATHWAY	MSigDB C2 CP	5	1
BIOCARTA_THELPER_PATHWAY	MSigDB C2 CP	5	1
CHROMOSOMEPERICENTRIC_REGION	MSigDB C5	5	1
CLASPER_LYMPHATIC_VESSELS_DURING_METASTASIS	MSigDB C2 CGP	5	1
E2F2_TARGETS	ACSN	5	1
GCM_PFN1	MSigDB C4	5	1
GNF2_NS	MSigDB C4	5	1
GNF2_SNRK	MSigDB C4	5	1
GNF2_XRCC5	MSigDB C4	5	1
GRATIAS_RETINOBLASTOMA_16Q24	MSigDB C2 CGP	5	1
GSE13547_2H_VS_12_H_ANTI_IGM_STIM_BCELL	MSigDB C7	5	1
GSE14415_NATURAL_TREG_VS_TCONV	MSigDB C7	5	1
GSE22886_CTRL_VS_LPS_24H_DC	MSigDB C7	5	1
GSE24634_IL4_VS_CTRL_TREATED_NAIVE_CD4_TCELL_DAY3	MSigDB C7	5	1
GSE24634_TEFF_VS_TCONV_DAY3_IN_CULTURE	MSigDB C7	5	1
GSE25088_WT_VS_STAT6_KO_MACROPHAGE	MSigDB C7	5	1
GSE29618_PDC_VS_MDC	MSigDB C7	5	1
GSE41176_UNSTIM_VS_ANTI_IGM_STIM_BCELL_1H	MSigDB C7	5	1
HALLMARK_MYC_TARGETS_V2	MSigDB H	5	1
MAPK_PI3K	Speed	5	1
MITOCHONDRIAL_MATRIX	MSigDB C5	5	1
MORF_DNMT1	MSigDB C4	5	1
MORF_GNB1	MSigDB C4	5	1
MORF_PCNA	MSigDB C4	5	1
MORF_RAC1	MSigDB C4	5	1
MORF_USP5	MSigDB C4	5	1
REACTOME_E2F_MEDIATED_REGULATION_OF_DNA_REPLICATION	MSigDB C2 CP	5	1
REACTOME_S_PHASE	MSigDB C2 CP	5	1
VERHAAK_GLIOBLASTOMA_MESENCHYMAL	MSigDB C2 CGP	5	1
ASTON_MAJOR_DEPRESSIVE_DISORDER	MSigDB C2 CGP	4	0
BIOCARTA_NKT_PATHWAY	MSigDB C2 CP	4	0
CELLULAR_DEFENSE_RESPONSE	MSigDB C5	4	0
CIT5_DN	CIT	4	0
E2F1_TARGETS	ACSN	4	0
FAELT_B_CLL_WITH_VH_REARRANGEMENTS	MSigDB C2 CGP	4	0
GASHIMA_NRG1_SIGLING	MSigDB C2 CGP	4	0
GNF2_MBD4	MSigDB C4	4	0
GRADE_COLON_VS_RECTAL_CANCER	MSigDB C2 CGP	4	0
GSE13484_UNSTIM_VS_YF17D_VACCINE_STIM_PBMC	MSigDB C7	4	0
GSE1432_1H_VS_24H_IFNG_MICROGLIA	MSigDB C7	4	0
GSE19198_CTRL_VS_IL21_TREATED_TCELL_6H	MSigDB C7	4	0
GSE22196_HEALTHY_VS_OBESE_MOUSE_SKIN_GAMMADELTA_TCELL	MSigDB C7	4	0
GSE23568_ID3_KO_VS_WT_CD8_TCELL	MSigDB C7	4	0
GSE24142_EARLY_THYMIC_PROGENITOR_VS2_THYMOCYTE_FETAL_UP	MSigDB C7	4	0
GSE24634_TEFF_VS_TCONV_DAY7_IN_CULTURE	MSigDB C7	4	0
GSE25085_FETAL_BM_VS_ADULT_BM_SP4_THYMIC_IMPLANT	MSigDB C7	4	0
GSE27241_WT_VS_RORGT_KO_TH17_POLARIZED_CD4_TCELL_TREATED_WITH_DIGOXIN	MSigDB C7	4	1
GSE29618_BCELL_VS_MDC	MSigDB C7	4	1
GSE29618_BCELL_VS_MONOCYTE_DAY7_FLU_VACCINE	MSigDB C7	4	1
GSE29618_MONOCYTE_VS_PDC_DAY7_FLU_VACCINE	MSigDB C7	4	0
GSE42021_TREG_PLN_VS_CD24INT_TREG_THYMUS	MSigDB C7	4	0
GSE7509_UNSTIM_VS_FCGRIIB_STIM_DC	MSigDB C7	4	1

GSE9988_LOW_LPS_VS_VEHICLE_TREATED_MONOCYTE	MSigDB C7	4	0
GSE9988_LPS_VS_VEHICLE_TREATED_MONOCYTE	MSigDB C7	4	0
HALLMARK_IL6_JAK_STAT3_SIGNALING	MSigDB H	4	0
HALMOS_CEBPA_TARGETS	MSigDB C2 CGP	4	0
JACKSONMT1_TARGETS	MSigDB C2 CGP	4	1
KEGG_TOLL_LIKE_RECEPTOR_SIGNALING_PATHWAY	MSigDB C2 CP	4	1
LEUKOCYTE_ACTIVATION	MSigDB C5	4	0
LIU_PROSTATE_CANCER	MSigDB C2 CGP	4	0
MITOCH_METABOLISM	ACSN	4	0
MODULE_169	MSigDB C4	4	0
MODULE_223	MSigDB C4	4	0
MODULE_64	MSigDB C4	4	1
MORF_MTA1	MSigDB C4	4	0
NADH_DEHYDROGENASE_COMPLEX	MSigDB C5	4	1
NIKOLSKY_BREAST_CANCER_19P13_AMPLICON	MSigDB C2 CGP	4	1
PID_CD8_TCR_DOWNSTREAM_PATHWAY	MSigDB C2 CP	4	1
PID_FOXM1_PATHWAY	MSigDB C2 CP	4	1
REACTOME_MEIOTIC_RECOMBINATION	MSigDB C2 CP	4	0
REACTOME_TRANSCRIPTION	MSigDB C2 CP	4	1
SENGUPTA_SOPHARYNGEAL_CARCINOMA	MSigDB C2 CGP	4	1
SPINDLE_CHECKPOINT	MSigDB C5	4	1
SPINDLE_POLE	MSigDB C5	4	1
ST_T_CELL_SIGNAL_TRANSDUCTION	MSigDB C2 CP	4	1
STAMBOLSKY_RESPONSE_TO_VITAMIN_D3	MSigDB C2 CGP	4	1
VALK_AML_CLUSTER_4	MSigDB C2 CGP	4	1
WHITFIELD_CELL_CYCLE_S	MSigDB C2 CGP	4	1
BIOCARTA_MCM_PATHWAY	MSigDB C2 CP	4	1
CAIRO_HEPATOBLASTOMA_CLASSES	MSigDB C2 CGP	4	1
CHEMELLO_SOLEUS_VS_EDL_MYOFIBERS	MSigDB C2 CGP	4	1
CIT4_DN	CIT	4	1
DACOSTA_UV_RESPONSE_VIA_ERCC3_COMMON	MSigDB C2 CGP	4	1
EXTRACELLULAR_MATRIX	MSigDB C5	4	1
GNF2_ANP32B	MSigDB C4	4	1
GNF2_CASP8	MSigDB C4	4	1
GNF2_HAT1	MSigDB C4	4	1
GNF2_PTX3	MSigDB C4	4	1
GSE14000_TRANSLATED_RNA_VS_MRNA_DC	MSigDB C7	4	1
GSE18791_UNSTIM_VS_NEWCATSLE_VIRUS_DC_6H	MSigDB C7	4	1
GSE21670_UNTREATED_VS_TGFB_IL6_TREATED_CD4_TCELL	MSigDB C7	4	1
GSE24634_TEFF_VS_TCONV_DAY10_IN_CULTURE	MSigDB C7	4	1
GSE36476_CTRL_VS_TSST_ACT_72H_MEMORY_CD4_TCELL_YOUNG	MSigDB C7	4	1
GSE39110_DAY3_VS_DAY6_POST_IMMUNIZATION_CD8_TCELL	MSigDB C7	4	1
GSE3920_UNTREATED_VS_IFNA_TREATED_ENDOTHELIAL_CELL	MSigDB C7	4	1
GSE41867_NAIVE_VS_DAY30_LCMV_ARMSTRONG_MEMORY_CD8_TCELL	MSigDB C7	4	1
GSE42021_CD24HI_VS_CD24INT_TREG_THYMUS	MSigDB C7	4	1
KEGG_ASTHMA	MSigDB C2 CP	4	1
KEGG_PRIMARY_IMMUNODEFICIENCY	MSigDB C2 CP	4	1
KEGG_T_CELL_RECEPTOR_SIGNALING_PATHWAY	MSigDB C2 CP	4	1
MODULE_5	MSigDB C4	4	1
MORF_DDB1	MSigDB C4	4	1
MORF_GMPS	MSigDB C4	4	1
MORF_MBD4	MSigDB C4	4	1
MORF_PPP1CC	MSigDB C4	4	1
MORF_PTPN11	MSigDB C4	4	1
MORF_RAB1A	MSigDB C4	4	1
MORI_LARGE_PRE_BII_LYMPHOCYTE	MSigDB C2 CGP	4	1
PENG_RAPAMYCIN_RESPONSE	MSigDB C2 CGP	4	1
PID_FANCONI_PATHWAY	MSigDB C2 CP	4	1
PROTEINACEOUS_EXTRACELLULAR_MATRIX	MSigDB C5	4	1
REACTOME_APC_C_CDC20_MEDIATED_DEGRADATION_OF_MITOTIC_PROTEINS	MSigDB C2 CP	4	1
REACTOME_CELL_CYCLE_CHECKPOINTS	MSigDB C2 CP	4	1
REACTOME_SMOOTH_MUSCLE_CONTRACTION	MSigDB C2 CP	4	1
BOYLAN_MULTIPLE_MYELOMA_C_D	MSigDB C2 CGP	3	0
BURTON_ADIPOGENESIS_7	MSigDB C2 CGP	3	0
CHNG_MULTIPLE_MYELOMA_HYPERPLOID	MSigDB C2 CGP	3	0
chr6p22	MSigDB C1	3	0
CIT11_DN	CIT	3	0
DING_LUNG_CANCER_MUTATED_FREQUENTLY	MSigDB C2 CGP	3	0
FURUKAWA_DUSP6_TARGETS_PCI35	MSigDB C2 CGP	3	0
GCM_ANP32B	MSigDB C4	3	0
GNF2_TTN	MSigDB C4	3	0
GSE11057_CD4_EFF_MEM_VS_PBMC	MSigDB C7	3	0
GSE1432_CTRL_VS_IFNG_24H_MICROGLIA	MSigDB C7	3	0
GSE1432_CTRL_VS_IFNG_6H_MICROGLIA	MSigDB C7	3	0
GSE1460_DP_VS_CD4_THYMOCYTE	MSigDB C7	3	0
GSE19198_1H_VS_24H_IL21_TREATED_TCELL	MSigDB C7	3	0
GSE19401_UNSTIM_VS_RETINOIC_ACID_AND_PAM2CSK4_STIM_FOLLICULAR_DC	MSigDB C7	3	0
GSE21380_NON_TFH_VS_TFH_CD4_TCELL	MSigDB C7	3	0
GSE22886_NAIVE_TCELL_VS_DC	MSigDB C7	3	0
GSE23568_ID3_TRANSDUCED_VS_ID3_KO_CD8_TCELL	MSigDB C7	3	0
GSE24634_IL4_VS_CTRL_TREATED_NAIVE_CD4_TCELL_DAY5	MSigDB C7	3	0
GSE25085_FETAL_LIVER_VS_ADULT_BM_SP4_THYMIC_IMPLANT	MSigDB C7	3	0
GSE25123_CTRL_VS_ROSIGLITAZONE_STIM_PPARG_KO_MACROPHAGE	MSigDB C7	3	1
GSE3039_CD4_TCELL_VS_B1_BCELL	MSigDB C7	3	0
GSE31082_VS_DP_THYMOCYTE	MSigDB C7	3	0
GSE34156_NOD2_LIGAND_VS_TLR1_TLR2_LIGAND_6H_TREATED_MONOCYTE	MSigDB C7	3	0
GSE34156_UNTREATED_VS_6H_TLR1_TLR2_LIGAND_TREATED_MONOCYTE	MSigDB C7	3	0
GSE39556_UNTREATED_VS_3H_POLYIC_INJ_MOUSE_CD8A_DC	MSigDB C7	3	0
GSE44649_NAIVE_VS_ACTIVATED_CD8_TCELL	MSigDB C7	3	0
GSE45365_NK_CELL_VS_CD11B_DC	MSigDB C7	3	0
GSE45365_WT_VS_IFNAR_KO_BCELL	MSigDB C7	3	0
GSE45739_UNSTIM_VS_ACD3_ACD28_STIM_NRAS_KO_CD4_TCELL	MSigDB C7	3	0
GSE7219_WT_VS_NIK_NFKB2_KO_DC	MSigDB C7	3	1
GSE9006_TYPE_1_VS_TYPE_2_DIABETES_PBMC_AT_DX	MSigDB C7	3	1
GSE9988_ANTI_TREM1_VS_ANTI_TREM1_AND_LPS_MONOCYTE	MSigDB C7	3	1
GUO_HEX_TARGETS	MSigDB C2 CGP	3	0
HISTONE_ACETYLTRANSFERASE_ACTIVITY	MSigDB C2 CGP	3	0
KIM_ALL_DISORDERS_OLIGODENDROCYTE_NUMBER_CORR	MSigDB C2 CGP	3	0
LI_INDUCED_T_TO_TURAL_KILLER	MSigDB C2 CGP	3	0
LOCOMOTORY_BEHAVIOR	MSigDB C5	3	0
LUI_THYROID_CANCER_PAX8_PPARG	MSigDB C2 CGP	3	0
MITOTIC_SISTER_CHROMATID_SEGREGATION	MSigDB C5	3	0
MONTERO_THYROID_CANCER_POOR_SURVIVAL	MSigDB C2 CGP	3	1
MORF_BAG5	MSigDB C4	3	1
OSMAN_BLADDER_CANCER	MSigDB C2 CGP	3	1
REACTOME_ACTIVATION_OF_NF_KAPPAB_IN_B_CELLS	MSigDB C2 CP	3	0
REACTOME_APC_C_CDH1_MEDIATED_DEGRADATION_OF_CDC20_AND_OTHER_APC_C_CDH1_TARGETED_PROTEINS_IN_LATE_MITOSIS_EARLY_G1	MSigDB C2 CP	3	0
REACTOME_CYCLIN_A_B1_ASSOCIATED_EVENTS_DURING_G2_M_TRANSITION	MSigDB C2 CP	3	0
REACTOME_CYTOKINE_SIGNALING_IN_IMMUNE_SYSTEM	MSigDB C2 CP	3	0
REACTOME_IL_3_5_AND_GM_CSF_SIGNALING	MSigDB C2 CP	3	0
REACTOME_RNA_POL_I_TRANSCRIPTION	MSigDB C2 CP	3	1
REACTOME_SCFSKP2_MEDIATED_DEGRADATION_OF_P27_P21	MSigDB C2 CP	3	1
REACTOME_STRIATED_MUSCLE_CONTRACTION	MSigDB C2 CP	3	1
REGULATION_OF_IMMUNE_SYSTEM_PROCESS	MSigDB C5	3	1
REGULATION_OF_MITOSIS	MSigDB C5	3	1
RESPIRATORY_CHAIN_COMPLEX_I	MSigDB C5	3	1
RHEIN_ALL_GLUCOCORTICOID_THERAPY	MSigDB C2 CGP	3	1
ROETH_TERT_TARGETS	MSigDB C2 CGP	3	0
TAYLOR_METHYLATED_IN_ACUTE_LYMPHOBLASTIC_LEUKEMIA	MSigDB C2 CGP	3	1
TIEN_INTESTINE_PROBIOTICS_24HR	MSigDB C2 CGP	3	1
TONKS_TARGETS_OF_RUNX1_RUNX1T1_FUSION_HSC	MSigDB C2 CGP	3	1
V$NRSF_01	MSigDB C3	3	1
VERHAAK_AML_WITH_NPM1_MUTATED	MSigDB C2 CGP	3	1
WANG_CLIM2_TARGETS	MSigDB C2 CGP	3	1
WANG_RESPONSE_TO_GSK3_INHIBITOR_SB216763	MSigDB C2 CGP	3	1
WANG_SMARCE1_TARGETS	MSigDB C2 CGP	3	1
WUNDER_INFLAMMATORY_RESPONSE_AND_CHOLESTEROL	MSigDB C2 CGP	3	0
ADAPTIVE_IMMUNE_RESPONSE	MSigDB C5	3	1

ADAPTIVE_IMMUNE_RESPONSE_GO_0002460	MSigDB C5	3	1
BIOCARTA_CSK_PATHWAY	MSigDB C2 CP	3	1
BIOCARTA_TCRA_PATHWAY	MSigDB C2 CP	3	1
CELL_CYCLE_GO_0007049	MSigDB C5	3	1
chr16p13	MSigDB C1	3	1
CHROMOSOMAL_PART	MSigDB C5	3	1
DNA_REPLICATION	MSigDB C5	3	1
FEVR_CTNNB1_TARGETS	MSigDB C2 CGP	3	1
GCM_DFFA	MSigDB C4	3	1
GCM_RAB10	MSigDB C4	3	1
GCM_UBE2N	MSigDB C4	3	1
GINESTIER_BREAST_CANCER_ZNF217_AMPLIFIED	MSigDB C2 CGP	3	1
GNF2_MLH1	MSigDB C4	3	1
GRAHAM_CML_DIVIDING_VS_NORMAL_QUIESCENT	MSigDB C2 CGP	3	1
GSE13485_CTRL_VS_DAY3_YF17D_VACCINE_PBMC	MSigDB C7	3	1
GSE21063_WT_VS_NFATC1_KO_3H_ANTI_IGM_STIM_BCELL	MSigDB C7	3	1
GSE2405_0H_VS_9H_A_PHAGOCYTOPHILUM_STIM_NEUTROPHIL	MSigDB C7	3	1
GSE24634_TREG_VS_TCONV_POST_DAY3_IL4_CONVERSION	MSigDB C7	3	1
GSE30971_CTRL_VS_LPS_STIM_MACROPHAGE_WBP7_KO_2H	MSigDB C7	3	1
GSE36476_CTRL_VS_TSST_ACT_40H_MEMORY_CD4_TCELL_OLD	MSigDB C7	3	1
GSE36476_CTRL_VS_TSST_ACT_72H_MEMORY_CD4_TCELL_OLD	MSigDB C7	3	1
GSE37533_PPARG2_FOXP3_VS_FOXP3_TRANSDUCED_CD4_TCELL	MSigDB C7	3	1
GSE42021_TREG_PLN_VS_TREG_PRECURSORS_THYMUS	MSigDB C7	3	1
GSE43863_DAY6_EFF_VS_DAY150_MEM_LY6C_INT_CXCR5POS_CD4_TCELL	MSigDB C7	3	1
GSE45837_WT_VS_GFI1_KO_PDC	MSigDB C7	3	1
GSE4984_UNTREATED_VS_GALECTIN1_TREATED_DC	MSigDB C7	3	1
GSE9988_ANTI_TREM1_VS_LOW_LPS_MONOCYTE	MSigDB C7	3	1
HALLMARK_COMPLEMENT	MSigDB H	3	1
HALLMARK_MYOGENESIS	MSigDB H	3	1
HELLER_SILENCED_BY_METHYLATION	MSigDB C2 CGP	3	1
JAEGER_METASTASIS	MSigDB C2 CGP	3	1
JECHLINGER_EPITHELIAL_TO_MESENCHYMAL_TRANSITION	MSigDB C2 CGP	3	1
KAYAMA_SOFT_TISSUE_TUMORS_PCA1	MSigDB C2 CGP	3	1
KEGG_DNA_REPLICATION	MSigDB C2 CP	3	1
LEE_BMP2_TARGETS	MSigDB C2 CGP	3	1
MITSIADES_RESPONSE_TO_APLIDIN	MSigDB C2 CGP	3	1
MODULE_119	MSigDB C4	3	1
MODULE_122	MSigDB C4	3	1
MODULE_329	MSigDB C4	3	1
MODULE_387	MSigDB C4	3	1
MODULE_451	MSigDB C4	3	1
MODULE_512	MSigDB C4	3	1
MODULE_98	MSigDB C4	3	1
MORF_DAP	MSigDB C4	3	1
MORF_MSH2	MSigDB C4	3	1
MORF_PPP2CA	MSigDB C4	3	1
MORF_PRKAG1	MSigDB C4	3	1
MORF_PRKDC	MSigDB C4	3	1
MORF_RAD54L	MSigDB C4	3	1
MORI_PRE_BI_LYMPHOCYTE	MSigDB C2 CGP	3	1
PUJA_BRCA_CENTERED_NETWORK	MSigDB C2 CGP	3	1
RCGCANGCGY_V$NRF1_Q6	MSigDB C3	3	1
REACTOME_ACTIVATION_OF_THE_MRNA_UPON_BINDING_OF_THE_CAP_BINDING_COMPLEX_AND_EIFS_AND_SUBSEQUENT_BINDING_TO_43S	MSigDB C2 CP	3	1
REACTOME_DOWNSTREAM_TCR_SIGNALING	MSigDB C2 CP	3	1
REACTOME_FORMATION_OF_THE_TERNARY_COMPLEX_AND_SUBSEQUENTLY_THE_43S_COMPLEX	MSigDB C2 CP	3	1
REACTOME_P53_DEPENDENT_G1_DNA_DAMAGE_RESPONSE	MSigDB C2 CP	3	1
REACTOME_PROCESSING_OF_CAPPED_INTRON_CONTAINING_PRE_MRNA	MSigDB C2 CP	3	1
REACTOME_REGULATION_OF_APOPTOSIS	MSigDB C2 CP	3	1
REACTOME_TRNA_AMINOACYLATION	MSigDB C2 CP	3	1
REN_ALVEOLAR_RHABDOMYOSARCOMA	MSigDB C2 CGP	3	1
RICKMAN_TUMOR_DIFFERENTIATED_WELL_VS_POORLY	MSigDB C2 CGP	3	1
ROSS_AML_OF_FAB_M7_TYPE	MSigDB C2 CGP	3	1
SCHLOSSER_MYC_AND_SERUM_RESPONSE_SYNERGY	MSigDB C2 CGP	3	1
SPLICEOSOME	MSigDB C5	3	1
STRUCTURAL_CONSTITUENT_OF_MUSCLE	MSigDB C5	3	1
TAKEDA_TARGETS_OF_NUP98_HOXA9_FUSION_10D	MSigDB C2 CGP	3	1
V$ELK1_02	MSigDB C3	3	1
V$NRF2_01	MSigDB C3	3	1
VECCHI_GASTRIC_CANCER_ADVANCED_VS_EARLY	MSigDB C2 CGP	3	1
ACCAATC,MIR-509	MSigDB C3	2	1
AIGNER_ZEB1_TARGETS	MSigDB C2 CGP	2	0
AMIT_SERUM_RESPONSE_480_MCF10A	MSigDB C2 CGP	2	0
BALDWIN_PRKCI_TARGETS	MSigDB C2 CGP	2	0
BIOCARTA_TH1TH2_PATHWAY	MSigDB C2 CP	2	0
CAHOY_NEURONAL	MSigDB C2 CGP	2	0
CAIRO_HEPATOBLASTOMA	MSigDB C2 CGP	2	0
CAR_HPX	MSigDB C4	2	0
CAR_IGFBP1	MSigDB C4	2	0
CELL_ACTIVATION	MSigDB C5	2	0
CHIANG_LIVER_CANCER_SUBCLASS_CTNNB1	MSigDB C2 CGP	2	0
CHIBA_RESPONSE_TO_TSA	MSigDB C2 CGP	2	0
chr17q25	MSigDB C1	2	0
COLDREN_GEFITINIB_RESISTANCE	MSigDB C2 CGP	2	0
CREIGHTON_AKT1_SIGLING_VIA_MTOR	MSigDB C2 CGP	2	0
CUI_TCF21_TARGETS	MSigDB C2 CGP	2	0
DAIRKEE_CANCER_PRONE_RESPONSE_E2	MSigDB C2 CGP	2	0
DAUER_STAT3_TARGETS	MSigDB C2 CGP	2	0
DELASER_MYOD_TARGETS	MSigDB C2 CGP	2	0
DELYS_THYROID_CANCER	MSigDB C2 CGP	2	0
DNA_METABOLIC_PROCESS	MSigDB C5	2	0
EBAUER_MYOGENIC_TARGETS_OF_PAX3_FOXO1_FUSION	MSigDB C2 CGP	2	0
FAELT_B_CLL_WITH_VH3_21	MSigDB C2 CGP	2	0
GAVIN_FOXP3_TARGETS_CLUSTER_P4	MSigDB C2 CGP	2	0
GCM_BAG5	MSigDB C4	2	0
GCM_CSNK1A1	MSigDB C4	2	0
GCNP_SHH_UP_LATE.V1_UP	MSigDB C6	2	0
GENTILE_UV_HIGH_DOSE	MSigDB C2 CGP	2	0
GENTILE_UV_RESPONSE_CLUSTER_D4	MSigDB C2 CGP	2	0
GGAANCGGAANY_UNKNOWN	MSigDB C3	2	0
GNF2_CEBPA	MSigDB C4	2	0
GNF2_CYP2B6	MSigDB C4	2	0
GNF2_DNM1	MSigDB C4	2	0
GNF2_GSTM1	MSigDB C4	2	0
GNF2_HPN	MSigDB C4	2	0
GNF2_HPX	MSigDB C4	2	0
GNF2_JAK1	MSigDB C4	2	0
GNF2_LCAT	MSigDB C4	2	0
GNF2_LYN	MSigDB C4	2	0
GNF2_MYL2	MSigDB C4	2	0
GNF2_MYL3	MSigDB C4	2	0
GNF2_SERPINI2	MSigDB C4	2	0
GNF2_TST	MSigDB C4	2	0
GOLDRATH_NAIVE_VS_MEMORY_CD8_TCELL	MSigDB C7	2	0
GSE10325_CD4_TCELL_VS_LUPUS_CD4_TCELL	MSigDB C7	2	0
GSE11057_CD4_CENT_MEM_VS_PBMC	MSigDB C7	2	0
GSE16266_LPS_VS_HEATSHOCK_AND_LPS_STIM_MEF	MSigDB C7	2	0
GSE17974_IL4_AND_ANTI_IL12_VS_UNTREATED_72H_ACT_CD4_TCELL	MSigDB C7	2	0
GSE18791_CTRL_VS_NEWCASTLE_VIRUS_DC_10H	MSigDB C7	2	0
GSE18791_CTRL_VS_NEWCASTLE_VIRUS_DC_6H	MSigDB C7	2	0
GSE18791_UNSTIM_VS_NEWCATSLE_VIRUS_DC_10H	MSigDB C7	2	0
GSE19401_NAIVE_VS_IMMUNIZED_MOUSE_PLN_FOLLICULAR_DC	MSigDB C7	2	0
GSE19888_ADENOSINE_A3R_INH_VS_ACT_WITH_INHIBITOR_PRETREATMENT_IN_MAST_CELL	MSigDB C7	2	0
GSE2128_CTRL_VS_MIMETOPE_NEGATIVE_SELECTION_DP_THYMOCYTE_NOD	MSigDB C7	2	0
GSE21360_SECONDARY_VS_QUATERNARY_MEMORY_CD8_TCELL	MSigDB C7	2	0
GSE21546_ELK1_KO_VS_SAP1A_KO_AND_ELK1_KO_DP_THYMOCYTES	MSigDB C7	2	0
GSE21670_STAT3_KO_VS_WT_CD4_TCELL_IL6_TREATED_UP	MSigDB C7	2	0
GSE21670_UNTREATED_VS_IL6_TREATED_STAT3_KO_CD4_TCELL	MSigDB C7	2	0
GSE22140_HEALTHY_VS_ARTHRITIC_MOUSE_CD4_TCELL	MSigDB C7	2	0

GSE22601_IMMATURE_CD4_SINGLE_POSITIVE_VS_CD8_SINGLE_POSITIVE_THYMOCYTE	MSigDB C7	2	0
GSE22886_CD4_TCELL_VS_BCELL_NAIVE	MSigDB C7	2	0
GSE22886_CD8_TCELL_VS_BCELL_NAIVE	MSigDB C7	2	0
GSE22886_DAY0_VS_DAY1_MONOCYTE_IN_CULTURE	MSigDB C7	2	0
GSE22886_DC_VS_MONOCYTE	MSigDB C7	2	0
GSE22886_NAIVE_BCELL_VS_MONOCYTE	MSigDB C7	2	0
GSE22886_TCELL_VS_BCELL_NAIVE	MSigDB C7	2	0
GSE22935_WT_VS_MYD88_KO_MACROPHAGE	MSigDB C7	2	0
GSE23568_CTRL_VS_ID3_TRANSDUCED_CD8_TCELL	MSigDB C7	2	0
GSE24634_TREG_VS_TCONV_POST_DAY5_IL4_CONVERSION	MSigDB C7	2	0
GSE26030_TH1_VS_TH17_DAY5_POST_POLARIZATION	MSigDB C7	2	1
GSE26343_UNSTIM_VS_LPS_STIM_NFAT5_KO_MACROPHAGE	MSigDB C7	2	1
GSE26495_NAIVE_VS_PD1LOW_CD8_TCELL	MSigDB C7	2	1
GSE29618_BCELL_VS_PDC	MSigDB C7	2	0
GSE29618_BCELL_VS_PDC_DAY7_FLU_VACCINE	MSigDB C7	2	1
GSE30971_CTRL_VS_LPS_STIM_MACROPHAGE_WBP7_KO_4H	MSigDB C7	2	0
GSE32164_RESTING_DIFFERENTIATED_VS_ALTERNATIVELY_ACT_M2_MACROPHAGE	MSigDB C7	2	0
GSE32533_MIR17_KO_VS_MIR17_OVEREXPRESS_ACT_CD4_TCELL	MSigDB C7	2	0
GSE34006_A2AR_KO_VS_A2AR_AGONIST_TREATED_TREG	MSigDB C7	2	0
GSE34156_TLR1_TLR2_LIGAND_VS_NOD2_AND_TLR1_TLR2_LIGAND_24H_TREATED_MONOCYTE	MSigDB C7	2	0
GSE36527_CD69_NEG_VS_POS_TREG_CD62L_LOS_KLRG1_NEG	MSigDB C7	2	0
GSE36888_UNTREATED_VS_IL2_TREATED_TCELL_6H	MSigDB C7	2	0
GSE41087_WT_VS_FOXP3_MUT_ANTI_CD3_CD28_STIM_CD4_TCELL	MSigDB C7	2	0
GSE41176_WT_VS_TAK1_KO_ANTI_IGM_STIM_BCELL_24H	MSigDB C7	2	0
GSE41867_NAIVE_VS_DAY15_LCMV_EFFECTOR_CD8_TCELL	MSigDB C7	2	0
GSE42021_TREG_VS_TCONV_PLN	MSigDB C7	2	0
GSE43863_DAY6_EFF_VS_DAY150_MEM_TH1_CD4_TCELL	MSigDB C7	2	0
GSE5099_CLASSICAL_M1_VS_ALTERNATIVE_M2_MACROPHAGE	MSigDB C7	2	0
GSE7509_DC_VS_MONOCYTE	MSigDB C7	2	1
GSE7768_OVA_ALONE_VS_OVA_WITH_LPS_IMMUNIZED_MOUSE_WHOLE_SPLEEN_6H	MSigDB C7	2	1
GSE8835_CD4_VS_CD8_TCELL_CLL_PATIENT	MSigDB C7	2	1
GSE9988_ANTI_TREM1_VS_CTRL_TREATED_MONOCYTES	MSigDB C7	2	1
GSE9988_ANTI_TREM1_VS_VEHICLE_TREATED_MONOCYTES	MSigDB C7	2	0
GSE9988_LOW_LPS_VS_CTRL_TREATED_MONOCYTE	MSigDB C7	2	0
GSE9988_LPS_VS_CTRL_TREATED_MONOCYTE	MSigDB C7	2	0
HADDAD_T_LYMPHOCYTE_AND_NK_PROGENITOR	MSigDB C2 CGP	2	0
HALLMARK_MTORC1_SIGNALING	MSigDB H	2	0
HOEGERKORP_CD44_TARGETS_DIRECT	MSigDB C2 CGP	2	1
HOEGERKORP_CD44_TARGETS_TEMPORAL	MSigDB C2 CGP	2	1
HOLLEMAN_VINCRISTINE_RESISTANCE_B_ALL	MSigDB C2 CGP	2	1
HORIUCHI_WTAP_TARGETS	MSigDB C2 CGP	2	1
HOXA9_DN.V1	MSigDB C2 CGP	2	1
INFLAMMATORY_RESPONSE	MSigDB C5	2	1
KEGG_VALINE_LEUCINE_AND_ISOLEUCINE_DEGRADATION	MSigDB C2 CP	2	1
KEGG_VIRAL_MYOCARDITIS	MSigDB C2 CP	2	0
KIM_ALL_DISORDERS_DURATION_CORR	MSigDB C2 CGP	2	0
LEE_LIVER_CANCER	MSigDB C2 CGP	2	0
LEE_LIVER_CANCER_SURVIVAL	MSigDB C2 CGP	2	0
LIN_APC_TARGETS	MSigDB C2 CGP	2	0
LINDGREN_BLADDER_CANCER_CLUSTER_1	MSigDB C2 CGP	2	0
LUI_THYROID_CANCER_CLUSTER_3	MSigDB C2 CGP	2	0
MA_MYELOID_DIFFERENTIATION	MSigDB C2 CGP	2	0
MARSON_FOXP3_TARGETS_STIMULATED	MSigDB C2 CGP	2	0
MITOTIC_CELL_CYCLE_CHECKPOINT	MSigDB C5	2	0
MODULE_117	MSigDB C4	2	0
MODULE_149	MSigDB C4	2	0
MODULE_15	MSigDB C4	2	0
MODULE_177	MSigDB C4	2	0
MODULE_201	MSigDB C4	2	0
MODULE_202	MSigDB C4	2	0
MODULE_212	MSigDB C4	2	0
MODULE_23	MSigDB C4	2	1
MODULE_397	MSigDB C4	2	1
MODULE_484	MSigDB C4	2	1
MORF_REV3L	MSigDB C4	2	0
MORF_TPR	MSigDB C4	2	0
MORI_MATURE_B_LYMPHOCYTE	MSigDB C2 CGP	2	1
MULLIGHAN_MLL_SIGTURE_1	MSigDB C2 CGP	2	1
MYOSIN_COMPLEX	MSigDB C5	2	1
NIELSEN_MALIGT_FIBROUS_HISTIOCYTOMA	MSigDB C2 CGP	2	1
NIKOLSKY_BREAST_CANCER_15Q26_AMPLICON	MSigDB C2 CGP	2	1
ORGANELLE_INNER_MEMBRANE	MSigDB C5	2	1
OXFORD_RALA_OR_RALB_TARGETS	MSigDB C2 CGP	2	1
PARK_APL_PATHOGENESIS	MSigDB C2 CGP	2	1
PARK_TRETINOIN_RESPONSE_AND_PML_RARA_FUSION	MSigDB C2 CGP	2	1
PATIL_LIVER_CANCER	MSigDB C2 CGP	2	1
PELLICCIOTTA_HDAC_IN_ANTIGEN_PRESENTATION	MSigDB C2 CGP	2	1
PHONG_TNF_TARGETS	MSigDB C2 CGP	2	1
PID_CXCR4_PATHWAY	MSigDB C2 CP	2	1
PLASARI_TGFB1_TARGETS_10HR	MSigDB C2 CGP	2	1
RADAEVA_RESPONSE_TO_IF1	MSigDB C2 CGP	2	0
REACTOME_ABORTIVE_ELONGATION_OF_HIV1_TRANSCRIPT_IN_THE_ABSENCE_OF_TAT	MSigDB C2 CP	2	0
REACTOME_AMYLOIDS	MSigDB C2 CP	2	0
REACTOME_BIOLOGICAL_OXIDATIONS	MSigDB C2 CP	2	0
REACTOME_CHEMOKINE_RECEPTORS_BIND_CHEMOKINES	MSigDB C2 CP	2	0
REACTOME_CLASS_A1_RHODOPSIN_LIKE_RECEPTORS	MSigDB C2 CP	2	0
REACTOME_DEFENSINS	MSigDB C2 CP	2	0
REACTOME_ER_PHAGOSOME_PATHWAY	MSigDB C2 CP	2	0
REACTOME_GENERIC_TRANSCRIPTION_PATHWAY	MSigDB C2 CP	2	0
REACTOME_IL_2_SIGNALING	MSigDB C2 CP	2	0
REACTOME_INNATE_IMMUNE_SYSTEM	MSigDB C2 CP	2	0
REACTOME_NEURONAL_SYSTEM	MSigDB C2 CP	2	0
REACTOME_SIGNALING_BY_ILS	MSigDB C2 CP	2	1
RESPONSE_TO_EXTERNAL_STIMULUS	MSigDB C5	2	1
RICKMAN_TUMOR_DIFFERENTIATED_WELL_VS_MODERATELY	MSigDB C2 CGP	2	1
SABATES_COLORECTAL_ADENOMA	MSigDB C2 CGP	2	0
SANSOM_APC_TARGETS	MSigDB C2 CGP	2	1
SARCOMERE	MSigDB C5	2	1
SCHLINGEMANN_SKIN_CARCINOGENESIS_TPA	MSigDB C2 CGP	2	1
SCIAN_CELL_CYCLE_TARGETS_OF_TP53_AND_TP73	MSigDB C2 CGP	2	1
SONG_TARGETS_OF_IE86_CMV_PROTEIN	MSigDB C2 CGP	2	1
SWEET_KRAS_TARGETS	MSigDB C2 CGP	2	1
TENEDINI_MEGAKARYOCYTE_MARKERS	MSigDB C2 CGP	2	1
TGFB	Speed	2	1
TIAN_TNF_SIGLING_VIA_NFKB	MSigDB C2 CGP	2	1
TSENG_IRS1_TARGETS	MSigDB C2 CGP	2	1
V$GABP_B	MSigDB C3	2	1
WILCOX_RESPONSE_TO_PROGESTERONE	MSigDB C2 CGP	2	1
WU_HBX_TARGETS_1	MSigDB C2 CGP	2	0
XU_HGF_TARGETS_INDUCED_BY_AKT1_48HR	MSigDB C2 CGP	2	0
ZWANG_CLASS_2_TRANSIENTLY_INDUCED_BY_EGF	MSigDB C2 CGP	2	0
BLUM_RESPONSE_TO_SALIRASIB	MSigDB C2 CGP	2	1
BOQUEST_STEM_CELL	MSigDB C2 CGP	2	1
BOYAULT_LIVER_CANCER_SUBCLASS_G3	MSigDB C2 CGP	2	1
CASORELLI_ACUTE_PROMYELOCYTIC_LEUKEMIA	MSigDB C2 CGP	2	1
CHARAFE_BREAST_CANCER_BASAL_VS_MESENCHYMAL	MSigDB C2 CGP	2	1
CHIANG_LIVER_CANCER_SUBCLASS_PROLIFERATION	MSigDB C2 CGP	2	1
CHIANG_LIVER_CANCER_SUBCLASS_UNNOTATED	MSigDB C2 CGP	2	1
chr19p13	MSigDB C1	2	1
chr1q21	MSigDB C1	2	1
CHROMOSOME_SEGREGATION	MSigDB C5	2	1
chrxp11	MSigDB C1	2	1
chrxq28	MSigDB C1	2	1
CIT20_DN	CIT	2	1
CIT6_DN	CIT	2	1
DUTERTRE_ESTRADIOL_RESPONSE_24HR	MSigDB C2 CGP	2	1
FARMER_BREAST_CANCER_CLUSTER_4	MSigDB C2 CGP	2	1
FOURNIER_ACIR_DEVELOPMENT_LATE_2	MSigDB C2 CGP	2	1

FRASOR_RESPONSE_TO_SERM_OR_FULVESTRANT	MSigDB C2 CGP	2	1
GAUSSMANN_MLL_AF4_FUSION_TARGETS_E	MSigDB C2 CGP	2	1
GCCATNTTG_V$YY1_Q6	MSigDB C4	2	1
GCM_GSPT1	MSigDB C4	2	1
GCM_HBP1	MSigDB C4	2	1
GCM_MLL	MSigDB C4	2	1
GCM_MYST2	MSigDB C4	2	1
GCM_NF2	MSigDB C4	2	1
GNF2_CD48	MSigDB C4	2	1
GNF2_CDH3	MSigDB C4	2	1
GNF2_DEK	MSigDB C4	2	1
GNF2_DENR	MSigDB C4	2	1
GNF2_PPP6C	MSigDB C4	2	1
GNF2_SPI1	MSigDB C4	2	1
GNF2_SPINK1	MSigDB C4	2	1
GNF2_SPRR1B	MSigDB C4	2	1
GOLDRATH_EFF_VS_MEMORY_CD8_TCELL	MSigDB C7	2	1
GRAHAM_CML_QUIESCENT_VS_NORMAL_QUIESCENT	MSigDB C2 CGP	2	1
GSE11386_NAIVE_VS_MEMORY_BCELL	MSigDB C7	2	1
GSE13485_DAY1_VS_DAY7_YF17D_VACCINE_PBMC	MSigDB C7	2	1
GSE13485_PRE_VS_POST_YF17D_VACCINATION_PBMC	MSigDB C7	2	1
GSE13547_2H_VS_12_H_ANTI_IGM_STIM_ZFX_KO_BCELL	MSigDB C7	2	1
GSE17301_ACD3_ACD28_VS_ACD3_ACD28_AND_IFNA5_STIM_CD8_TCELL	MSigDB C7	2	1
GSE18791_CTRL_VS_NEWCASTLE_VIRUS_DC_8H	MSigDB C7	2	1
GSE18893_TCONV_VS_TREG_24H_TNF_STIM	MSigDB C7	2	1
GSE21546_WT_VS_SAP1A_KO_DP_THYMOCYTES	MSigDB C7	2	1
GSE22886_UNSTIM_VS_IL15_STIM_NKCELL	MSigDB C7	2	1
GSE22886_UNSTIM_VS_IL2_STIM_NKCELL	MSigDB C7	2	1
GSE2405_S_AUREUS_VS_UNTREATED_NEUTROPHIL	MSigDB C7	2	1
GSE29614_CTRL_VS_DAY7_TIV_FLU_VACCINE_PBMC	MSigDB C7	2	1
GSE3337_4H_VS_16H_IFNG_IN_CD8POS_DC	MSigDB C7	2	1
GSE36476_CTRL_VS_TSST_ACT_40H_MEMORY_CD4_TCELL_YOUNG	MSigDB C7	2	1
GSE36826_NORMAL_VS_STAPH_AUREUS_INF_IL1R_KO_SKIN	MSigDB C7	2	1
GSE40274_CTRL_VS_FOXP3_TRANSDUCED_ACTIVATED_CD4_TCELL	MSigDB C7	2	1
GSE45365_HEALTHY_VS_MCMV_INFECTION_CD11B_DC	MSigDB C7	2	1
GSE6259_33D1_POS_DC_VS_CD4_TCELL	MSigDB C7	2	1
GSE9988_ANTI_TREM1_VS_LPS_MONOCYTE	MSigDB C7	2	1
ICHIBA_GRAFT_VERSUS_HOST_DISEASE_35D	MSigDB C2 CGP	2	1
KAUFFMANN_D_REPLICATION_GENES	MSigDB C2 CGP	2	1
KEGG_AMINOACYL_TRNA_BIOSYNTHESIS	MSigDB C2 CP	2	1
KIM_GLIS2_TARGETS	MSigDB C2 CGP	2	1
LIEN_BREAST_CARCINOMA_METAPLASTIC	MSigDB C2 CGP	2	1
LIM_MAMMARY_LUMIL_MATURE	MSigDB C2 CGP	2	1
LINDGREN_BLADDER_CANCER_CLUSTER_3	MSigDB C2 CGP	2	1
LINDSTEDT_DENDRITIC_CELL_MATURATION_D	MSigDB C2 CGP	2	1
LIU_VAV3_PROSTATE_CARCINOGENESIS	MSigDB C2 CGP	2	1
MODULE_11	MSigDB C4	2	1
MODULE_12	MSigDB C4	2	1
MODULE_17	MSigDB C4	2	1
MODULE_2	MSigDB C4	2	1
MODULE_297	MSigDB C4	2	1
MODULE_357	MSigDB C4	2	1
MORF_BECN1	MSigDB C4	2	1
MORF_PAPSS1	MSigDB C4	2	1
MORF_SP3	MSigDB C4	2	1
NABA_ECM_GLYCOPROTEINS	MSigDB C2 CP	2	1
ONDER_CDH1_TARGETS_2	MSigDB C2 CGP	2	1
PENG_LEUCINE_DEPRIVATION	MSigDB C2 CGP	2	1
POMEROY_MEDULLOBLASTOMA_PROGNOSIS	MSigDB C2 CGP	2	1
PYEON_HPV_POSITIVE_TUMORS	MSigDB C2 CGP	2	1
REACTOME_LAGGING_STRAND_SYNTHESIS	MSigDB C2 CP	2	1
REACTOME_M_G1_TRANSITION	MSigDB C2 CP	2	1
REACTOME_MRNA_PROCESSING	MSigDB C2 CP	2	1
REACTOME_MUSCLE_CONTRACTION	MSigDB C2 CP	2	1
REACTOME_ORC1_REMOVAL_FROM_CHROMATIN	MSigDB C2 CP	2	1
REACTOME_PHOSPHORYLATION_OF_THE_APC_C	MSigDB C2 CP	2	1
REACTOME_SYNTHESIS_OF_DNA	MSigDB C2 CP	2	1
REACTOME_UNWINDING_OF_DNA	MSigDB C2 CP	2	1
SARRIO_EPITHELIAL_MESENCHYMAL_TRANSITION	MSigDB C2 CGP	2	1
SISTER_CHROMATID_SEGREGATION	MSigDB C5	2	1
SMALL_NUCLEAR_RIBONUCLEOPROTEIN_COMPLEX	MSigDB C5	2	1
SMID_BREAST_CANCER_LUMIL_B	MSigDB C2 CGP	2	1
WHITFIELD_CELL_CYCLE_G2_M	MSigDB C2 CGP	2	1

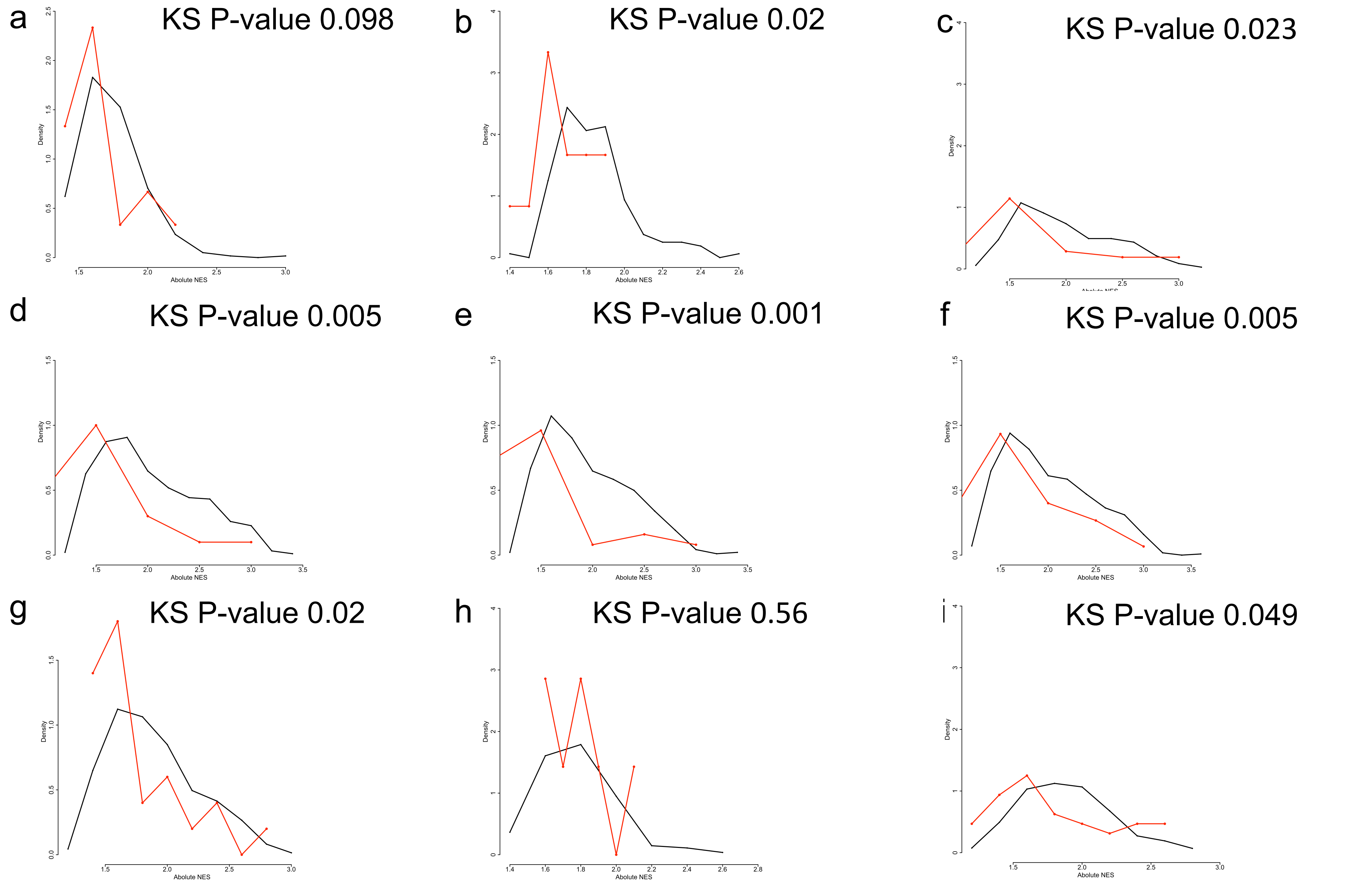

Figure S1. Comparison GSEA absolute NES values for the informative GSEA significant (black) vs. Hallmark GSEA significant (red) in (a) colorectal cancer KRAS mutated vs. wild type; (b-c) colon cancer metastatic vs. primary in RNA-seq and microarray; (d-g) tumor vs. normal in cervix, colon, gastric and lung and (h-i) breast cancer late vs early stage in TCGA and METABRIC, respectively.

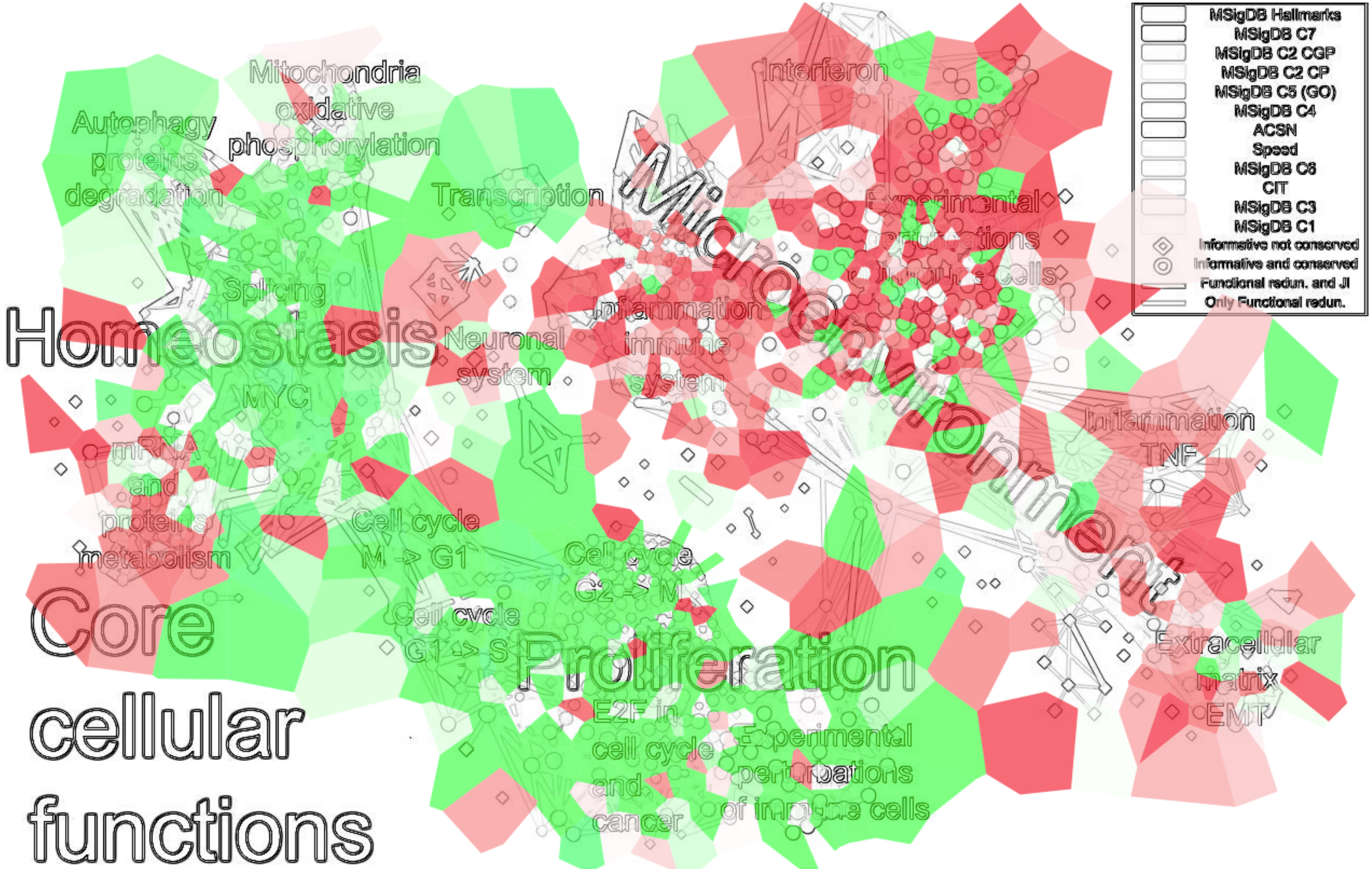

Figure S2. Fold changes of ROMA activity on the expression profiling of human CD4+ T cell during differentiation induction plotted on InfoSigMap. Red, and green indicate upregulation and downregulation, respectively.

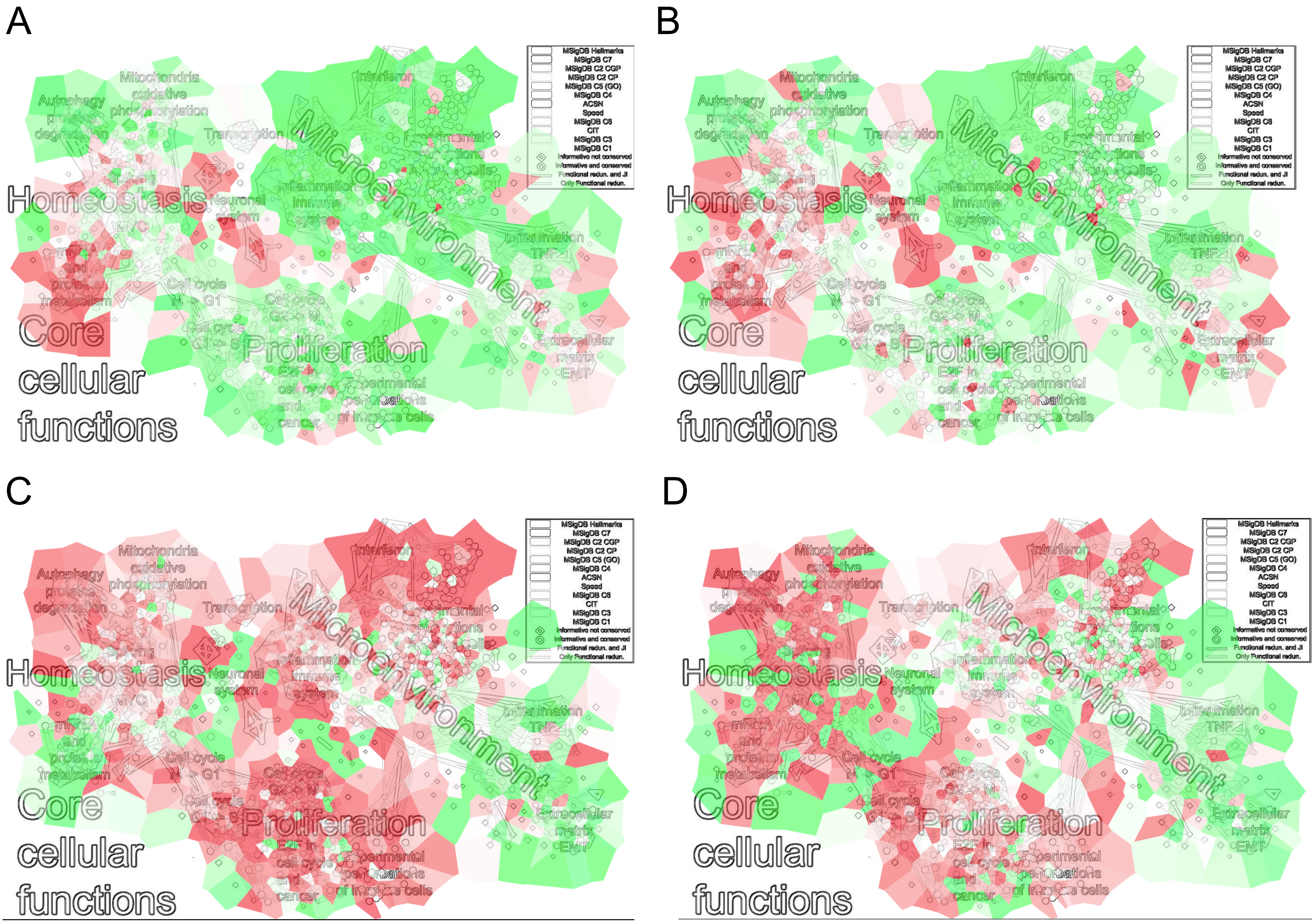

Figure S3. The significant fold-changes resulting from the differential ROMA analysis (see Methods) are plotted on the top of the map according to a heatmap coloring highlighting up- (red) and down-regulated (green) gene signatures. The plots are organized as follows : (a) colon cancer metastatic vs. primary in TCGA microarray; (a) colon cancer metastatic vs. primary in TCGA RNA-seq; (c) breast cancer late vs early stage in TCGA and (d) breast cancer late vs early in METABRIC.
